# Computational discovery of emodin-based anthraquinones as PARP-1 inhibitors with relevance to ovarian and prostate cancer

**DOI:** 10.1038/s41598-026-55381-4

**Published:** 2026-06-21

**Authors:** Sohag Ahmed, Md Reaz Uddin, Subrato Das, Abdulla All Maruf, Ahmed Nasim, Monotosh Kumar Sarkar, Shu Shanta, Rubel Hossain, Subash Chandra Shaha, Chinmoy Kumar Saha, Md Shafiqul Islam Sovon, Fahmida Akter, Abdullah Al Noman, Chetan Ashok, Srikanth Jeyabalan, Thakur Gurjeet Singh, Ling Shing Wong, Vetriselvan Subramaniyan

**Affiliations:** 1 C. Eugene Bennett Department of Chemistry, West Virginia University, Morgantown, WV 26506 USA; 2 Department of Pharmaceutical Technology, Faculty of Pharmacy, University of Dhaka, Dhaka, 1000 Bangladesh; 3 Department of Pharmaceutical Chemistry, Faculty of Pharmacy, University of Dhaka, Dhaka, 1000 Bangladesh; 4 Department of Pharmaceutical Technology, Faculty of Pharmacy, University of Asia Pacific, Dhaka, 1205 Bangladesh; 5 Department of Pharmacy, Faculty of Life Science, University of Development Alternative, Dhaka, 1209 Bangladesh; 6 Department of Chemistry, University of Louisiana at Lafayette, Lafayette, LA 70504 USA; 7 School of Public Health, The University of Memphis, Memphis, TN 38152 USA; 8 Department of Chemistry, University of Dhaka, Dhaka, 1000 Bangladesh; 9 Department of Biochemistry & Molecular Biology, Faculty of Science, University of Rajshahi, Rajshahi, 6205 Bangladesh; 10 Department of Pharmaceutical Sciences, North Dakota State University, Fargo, ND 58102 USA; 11 Department of Pharmacy, University of Chittagong, Chittagong University Road, Chittagong, 4331 Bangladesh; 12 School of Pharmacy, BRAC University, Dhaka, 1212 Bangladesh; 13 Department of Pharmacology, Faculty of Pharmacy, Sri Ramachandra Institute of Higher Education and Research (DU), Chennai, Tamil Nadu 600 116 India; 14 Chitkara College of Pharmacy, Chitkara University, Rajpura, Patiala, Punjab 140401 India; 15 Faculty of Health and Life Sciences, INTI International University, 71800 Nilai, Malaysia; 16 Faculty of Nursing, Shinawatra University, 99 Moo 10, Bangtoey, Samkhok, Pathum Thani 12160 Thailand; 17 Department of Biomedical Sciences, Sir Jeffrey Cheah Sunway Medical School, Faculty of Medical and Life Sciences, Sunway University, Petaling Jaya, Malaysia; 18 College of Health Sciences, Abu Dhabi University, Abu Dhabi, United Arab Emirates; 19 Global Research Cell, Dr. D. Y. Patil Dental College & Hospital, Dr. D. Y. Patil Vidyapeeth (Deemed to Be University), Pune, India; 20 Faculty of Health Sciences, Villa College Campus, Malé, Maldives 20373; 21 Department of Molecular and Cellular Biology, Kennesaw State University, Kennesaw, GA 30144 USA; 22 Global Research Fellow (GloRE), Faculty of Pharmacy and Health Sciences, Universiti Kuala Lumpur, Royal College of Medicine Perak (UniKL RCMP), No. 3, Jalan Greentown, Perak 30450 Ipoh, Malaysia

**Keywords:** Anticancer, Emodin, Molecular docking, ADMET, Molecular dynamics simulation, DCCM, And PCA, Cancer, Computational biology and bioinformatics, Drug discovery

## Abstract

Cancer is a disease characterized by genomic instability and aberrant DNA repair. Poly (ADP-ribose) polymerase-1 (PARP-1) represents a well-established therapeutic target, particularly in ovarian and prostate cancer. However, the currently approved PARP inhibitors face challenges such as resistance, toxicity, and reduced efficacy. The search for alternative scaffolds has therefore become increasingly urgent. In this study, we used an integrated approach combining computer-aided methods to search for potential lead compounds among emodin-based anthraquinone derivatives as PARP-1 inhibitors. Using a PASS-based QSAR approach, drug-likeness prediction, and in silico ADMET assessment, we pre-screened a large set of anthraquinones and identified several potential hits for interaction with PARP-1. These hits were studied using molecular docking with the PARP-1 catalytic domain (PDB ID: 7KK4). The most stable and compact complexes were further explored by 500 ns molecular dynamics (MD) simulations and various dynamic properties (RMSD, RMSF, Rg, SASA, MolSA, hydrogen bonds, PCA, DCCM). The key finding of this study is that several emodin-derived anthraquinones exhibited binding behavior and ADMET profiles comparable to, or better than, the reference PARP-1 inhibitor. Among them, CID-10425624 emerged as the most promising candidate, exhibiting stable binding, reduced conformational fluctuation, compact complex formation, persistent hydrogen-bond interactions, and enhanced dynamic residue correlations within the PARP-1 catalytic domain. These findings suggest that the anthraquinone scaffold can provide a valuable starting point for developing structurally distinct PARP-1 inhibitors. In summary, this study identified several emodin-derived anthraquinones, particularly CID-10425624, as computationally prioritized lead candidates for PARP-1 inhibition, providing a novel anthraquinone-based scaffold for further experimental validation and optimization.

**Supplementary Information:**

The online version contains supplementary material available at https://doi.org/10.1038/s41598-026-55381-4.

## Introduction

Genomic instability is considered a characteristic hallmark of cancer, and it forms the basis of tumor development, progression, and resistance to therapy. DNA repair proteins are often overexpressed, as malignant cells seek to take advantage of the defects in DNA damage response (DDR) pathways to allow uncontrolled proliferation^1^. Ovarian and prostate cancers are among the most relevant examples of solid tumors associated with unregulated DNA repair mechanisms, which negatively affect the disease progression and clinical outcomes^2^. Although therapeutic benefits have been made, resistance to treatment and relapses are the greatest issues, which have raised the necessity to develop new small-molecule inhibitors that can specifically target cancer-specific pathways for survival^3^.

Poly [ADP-ribose] polymerase-1 (PARP-1) is a hub mediator of double-strand break (DSB) repair, base excision repair, and single-strand DNA break repair. When DNA damage is sensed, PARP-1 catalyses poly-ADP ribosylation of itself and related repair proteins, which enables chromatin remodeling and repair complex recruitment^4^. Table 1 represents the role of PARP-1 in the progression of different cancers. The pharmacological inhibition of PARP-1 causes the formation of DNA damage and replication fork breakdown, which ultimately leads to cell death, especially in tumors with an inability to repair homologous recombination, such as BRCA-mutated ovarian and prostate cancers^5^. Despite a number of PARP-blockers that have been successfully used in clinical settings, their effects are short-lived due to the development of resistance, off-target toxicity, and lack of efficacy in tumor’s with no classical DDR mutations^6^. These restrictions underscore the need to find other chemical scaffolds that can inhibit PARP-1 with better selectivity and binding affinity^7^.

**Table 1:** Reported association of PARP-1 with different cancer types. The table summarizes representative literature reports describing the involvement of PARP-1 in various cancers, including bladder, breast, cervical, colon, colorectal, esophageal, gastric, liver, lung, oral, ovarian, pancreatic, prostate, and renal cancers. These references support the biological relevance of PARP-1 as a therapeutic target across multiple malignancies.

Type of cancer	List of standard article	Type of cancer	List of standard article
Bladder cancer	^113,114^	Liver cancer	^115–117^
Breast cancer	^118–120^	Lung cancer	^121–123^
Cervical cancer	^124–126^	Oral cancer	^127–129^
Colon cancer	^130–132^	Ovarian cancer	^133–135^
Colorectal cancer	^136–138^	Pancreatic cancer	^139–141^
Esophageal cancer	^142–144^	Prostate cancer	^145–147^
Gastric cancer	^148–150^	Renal cancer	^151–153^

Natural products are longstanding providers of structurally and biologically active compounds that have historically influenced the discovery of anticancer drugs^8–10^. Numerous anticancer drugs approved for clinical use are natural products or based on the natural product scaffolds because they are naturally compatible with biological targets^11^. In particular, anthraquinone derivatives have gained significant attention due to their planar aromatic cores, redox-reactive properties, and their capacity to bind to nucleic acids and protein active sites. These properties render anthraquinones promising candidates for inhibiting enzymes involved in DNA metabolism and repair^12^.

Emodin (1,3,8-trihydroxy-6-methylanthraquinone) is a naturally occurring anthraquinone that has been found in a number of medicinal plants, such as Rheum and Polygonum species^13^. Hsu and Chung (2012) along with Ma et al. (2021) have previously reported that emodin has antiproliferative and pro-apoptotic effects on a wide spectrum of cancer models that are mediated by the regulation of cell cycle regulators, oxidative stress, and DNA damage signaling networks^14^. Significantly, the chemical structure of emodin can be altered, and analogues with increased potency, selectivity or pharmacokinetic properties can be designed. There is currently emerging evidence that anthraquinone-based compounds could disrupt DNA repair enzymes, which positions emodin as a viable scaffold for PARP-1-targeted drug discovery^15^. Nevertheless, the molecular mechanism of emodin-PARP-1 interactions and dynamic stability of the corresponding complexes are poorly understood^16^.

While there are anthraquinone-based compounds reported to have PARP-1 inhibitory anticancer activities, these studies differ significantly from the current work in terms of depth of analysis. Earlier work by Lee et al. identified a suite of synthetic anthraquinone-derived small molecules that have antiproliferative effects in NSCLC cell lines and inhibit PARP-1 activity in vitro. Specifically, NSC747854 emerged as the most active compound in cell-based assays and possessed the greatest inhibitory activity against PARP-1. However, no detailed structure-based analysis of the PARP-1 catalytic domain with such compounds was performed^17^. Anthraquinone-based compounds such as emodin have been studied extensively as anticancer agents inducing apoptosis and/or cell cycle arrest through multiple signaling pathways. In leukemia and EBV-positive tumor cell lines as well as in primary cultures of human HCC cell lines, emodin was found to have radiosensitizing effects associated with enhanced PARP1 cleavage^18,19^. In this study, emodin and its analogues were screened for potential activity as PARP-1 inhibitors in the context of ovarian and prostate cancers. The findings from this work, which employed virtual screening followed by explicit molecular dynamics simulations, detailed binding free energy determinations and ADMET predictions, provided an in-depth characterization of the binding affinity and interaction patterns of ligands with PARP-1 in the active site, and revealed complex stability for lead compounds.

Molecular docking and molecular dynamics (MD) simulations, which represent computational approaches, are valuable tools that can be used to uncover the ligand–protein interaction mechanism and predict the binding stability at the atomic scale^20^. Docking can quickly screen and rank candidate molecules by predicted binding affinity and interaction patterns, whereas MD simulations can capture time-dependent changes in conformational changes, solvent effects, and interaction persistence that are not accessible through the use of simple models^21^. These tools, combined, provide a powerful strategy for identifying and optimizing lead compounds at an early stage before experimentation^22^.

In this study, we used a structure-based computational approach to study emodin and its analogs to identify potential PARP-1 inhibitors applicable to ovarian and prostate cancer. The molecular docking was used to select the ligands with good binding poses and interaction patterns in the PARP-1 catalytic site. The ligands that scored the highest, showing similar or even better binding affinity when compared with the standard inhibitor, were then subjected to molecular dynamics simulations to determine the stability of the complexes, their behavior, and the presence of important intermolecular interactions. This study is expected to provide mechanistic understanding of the inhibition of the PARP-1 by emodin-based scaffold and can be used in the rational design of new small-molecule inhibitors for targeted cancer therapy.

## Computational method and working procedure

This study is fully computational, and thus the methodology is presented as a computational pathway from ligand selection to activity prediction of activity, followed by docking, ADMET, molecular dynamics simulations, and analysis of the resulting trajectories for structural information.

### Online screening for ligand selection

In the early stage of drug discovery, computational screening can facilitate the process of compound selection, which exhibits positive physicochemical and biological characteristics at minimal experimentation cost and time^8,9,23^. The systematic in-silico screening strategy was used in this research to select emodin-based analogues, which have the potential to inhibit poly [ADP-ribose] polymerase-1 (PARP-1). The first pool of ligands was obtained by structural similarity to emodin and retaining the anthraquinone core of the ligand with functional group alternatives in order to enhance drug-like characteristics. The rule of five was first applied to measure the drug-like and oral bioavailability potential of all candidate compounds^24^. Compounds that broke more than one of the Lipinski criteria were ruled out to have a good molecular weight, the number of HB acceptors and donors, lipophilicity, and physicochemical suitability. Prediction of a biological activity of the filtered compounds was then done by the use of PASS analysis to determine the possibility of the compounds having anticancer and enzyme inhibitory activity of interest to DNA damage response pathways. To further improve the set of ligands, in silico ADMET profiling was carried out through Deep-PK (https://biosig.lab.uq.edu.au/deeppk/prediction) to analyze the absorption, distribution, metabolism, excretion, and toxicity properties^25–27^. This step made it possible to remove compounds whose intestinal absorption was predicted to be low, as well as those with poor metabolic stability, high toxicity, or poor bioavailability^28–30^. Compounds that had shown satisfactory ADMET profiles and predicted biological relevance were left to undergo downstream structure-based analysis^31–33^. The resulting subset of emodin analogues was further narrowed down to drug-like, predicted biological activity and pharmacokinetic suitable molecules to undergo a molecular docking analysis through Autodock Vina to determine their binding mechanisms and interaction potential within the catalytic site of PARP-1^34,35^. The results of docking and elaborate analyses of the interaction are given in a later section.

### Selection of protein

**Fig. 1:**
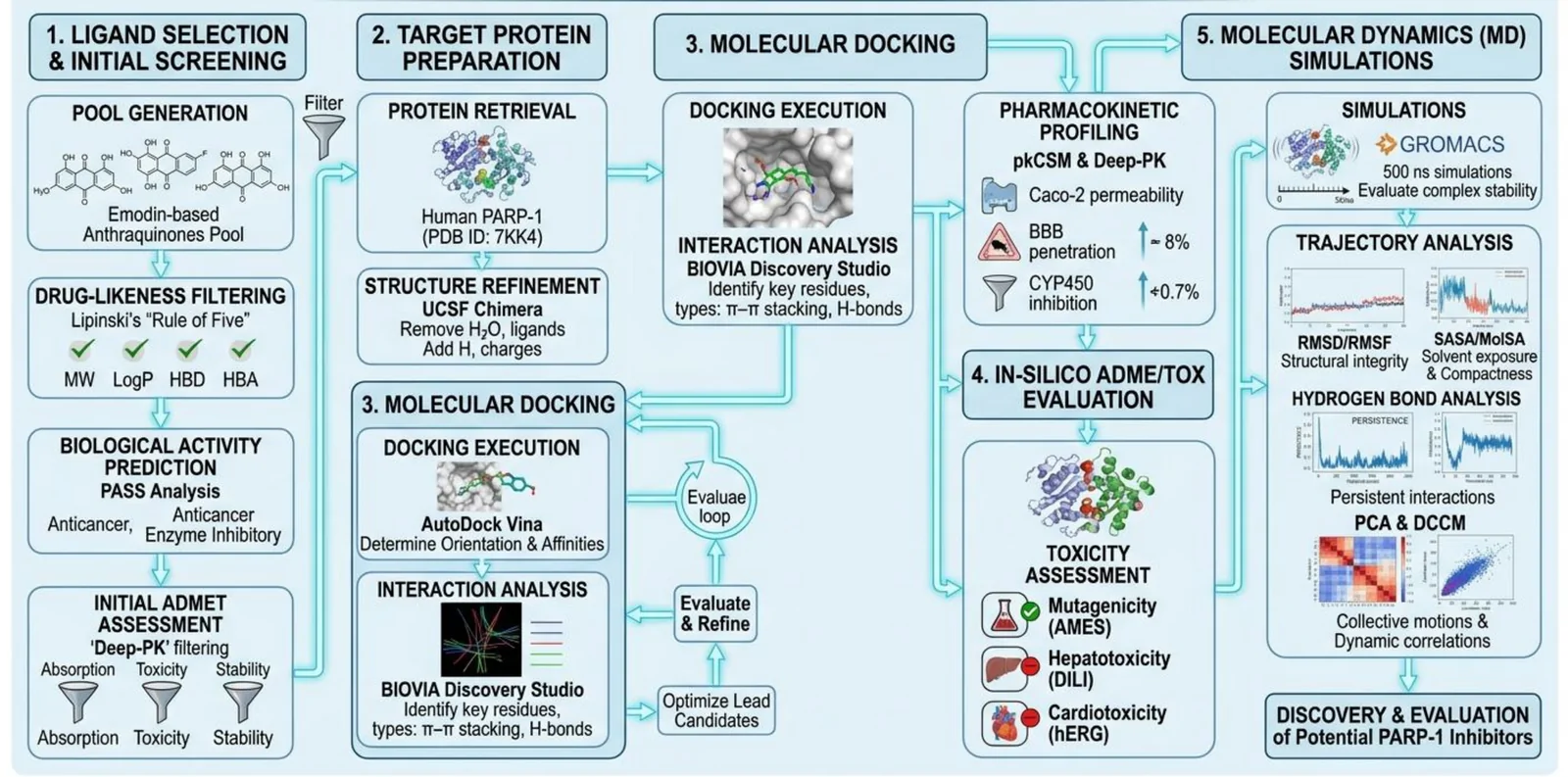
Schematic overview of the computational workflow used for identifying potential PARP-1 inhibitors. The workflow represents the sequential steps used in this study, including emodin-based ligand selection, drug-likeness and biological activity screening, PARP-1 structure preparation, molecular docking, ADMET/toxicity evaluation, 500 ns molecular dynamics simulation, trajectory analysis, and final discovery evaluation of potential PARP-1 inhibitor candidates.

A three-dimensional structure of human poly[ADP-ribose] polymerase-1 (PARP-1) utilized in the current study was retrieved from the Protein Data Bank (PDB, https://www.rcsb.org/)^36,37^ (Fig. 1). The crystal structure (PDB ID 7KK4) (Fig. S1) was chosen for molecular docking as it represents the catalytic domain of the PARP-1 co-crystallized with a small-molecule inhibitor, which gives a well-defined and experimentally verified binding pocket in the structure-based drug design. The high-resolution structure of the protein ensures reliable atomic coordinates, which are essential for better protein–ligand interaction modeling and molecular dynamics (MD) simulations^38^. Furthermore, the presence of a bound standard in the protein allowed a direct comparison of the binding poses of ligands, including emodin and its analogues, within the biologically relevant NAD⁺-binding site of PARP-1^39^. The protein structure was prepared in UCSF Chimera by removing co-crystallized water molecules and non-essential bound small molecules, followed by the addition of hydrogens and the assignment of charges prior to docking. No explicit missing-loop modeling was performed, as the selected catalytic-domain structure was considered suitable in its deposited form for docking and MD simulations^34,35,40^. The primary objective of the study is to investigate the binding mechanism and dynamics of the protein upon binding to emodin-based scaffolds at the PARP-1 catalytic site, the use of a single, high-quality structure was considered sufficient and appropriate for the docking and MD simulations^41^.

### Prediction of biological activity profiles

In silico biological activity prediction was performed using the Prediction of Activity Spectra for Substances (PASS) online platform (https://www.way2drug.com/passonline/) to prioritize emodin and its analogues for the structure-based studies^8^. PASS is a widely used QSAR-based tool that is used to predict the specific biological activity of chemical compounds based on their structural features and known structure–activity relationships. For the ligand-based approach, PASS was employed, since it permits rapid prioritization of structurally related compounds solely based on their chemical structures. For the screening of a set of emodin analogues, which vary in their substituent patterns, but have identical anthraquinone scaffold, PASS predicts a wide variety of activities, including those related to anticancer effects. Utilizing structural descriptors, PASS serves as a pre-docking filter to discriminate between compounds with higher probabilities for relevant biological activities, such as anticancer effects and enzyme inhibition. Thereafter, the identified promising hits can be subjected to target-based approaches (molecular docking) to also assess the binding to specific enzyme pockets^42–44^. The method provides two key parameters: the probability of activity (Pa) and the probability of inactivity (Pi), where the compounds with higher Pa and lower Pi are considered to exhibit the predicted biological activity. In this research, PASS analysis was employed to assess the likelihood of anticancer-relevant activities, including antineoplastic, antimutagenic, enzyme inhibitory, and redox-associated properties that are relevant to DNA damage response and repair pathways^45^. The compounds with potentially favorable Pa/Pi profiles within multiple cancer-related biological endpoints were considered biologically promising and taken for molecular docking and molecular dynamics simulation study^46^.

### Target protein–ligand preparation and molecular docking

Molecular docking is a widely used computational technique to evaluate protein–ligand interactions. Molecular docking was performed to predict the binding orientation and binding affinity of emodin and its analogues to the catalytic site of the poly[ADP-ribose] polymerase-1 (PARP-1)^47,48^. Structure-based docking is a rapid and cost-effective method for prioritizing ligands for further dynamics and experimental studies. AutoDock Vina was used in this study due to its validated scoring functions and widespread use in protein–ligand docking studies^49^.

The three-dimensional structure of the target protein was obtained from the Protein Data Bank (PDB ID: 7KK4)^50^. This human PARP-1 protein represents the catalytic domain co-crystallized with a small molecule inhibitor that provides a relevant and well-defined binding pocket suitable for docking analysis. In this study, the docking region was determined directly from the position of the co-crystallized ligand in the crystal structure of 7KK4, rather than from an external binding-site prediction tool such as CASTp or DoGSite. The co-crystallized ligand was first used to define the position of the center of the docking box and the size, followed by deletion of the ligand from the structure before performing the docking calculations on emodin and its analogues. The selected docking space includes the PARP-1 catalytic NAD+-binding/nicotinamide binding region within the catalytic domain. This region includes ligand recognition sites, in particular, Gly863, Ser904, Tyr907, Tyr889, His862, and Tyr896, and the neighboring residues in the catalytic pocket^50^. UCSF Chimera (Version 1.16) was used for the protein preparation, where the co-crystallized water and small molecules were removed from the protein^34,51^. The Dock Prep tool was used to add hydrogen and charges to the protein and saved as a Mol2 format for the docking. The preparation of the ligand was also carried out using the UCSF Chimera (Version 1.16) to ensure consistency with the receptor preparation protocol. The 3D SDF structure of the emodin scaffolds was downloaded from PubChem, and the structure energy was minimized and saved as mol2 for the docking. AutoDock Vina was used for the docking simulations with the UCSF Chimera graphical interface^35^. Docking calculations were performed by defining a grid box centered on the PARP-1 catalytic domain that was obtained from the crystal structure of the protein. The coordinates of the grid box were set to x = 22.7089, y = 5.3681, and z = 19.6233, and grid dimensions of 22.8984 × 22.3812 × 19.0542 Å^3^ along the x, y, and z axes, respectively. The grid parameters were selected to be able to adequately cover the NAD+-binding region, as well as the nearby residues engaged in ligand recognition, and provide enough conformational space to sample the flexibilities of the ligand. The receptor was fixed, and ligands were flexible, and docking simulations were performed with default AutoDock Vina parameters. The predicted binding free energy (kcal/mol) was used to rank binding poses, and the conformations scoring highest in the ranking were chosen to proceed to interaction analysis and further molecular dynamics simulations.

### ADMET study

To qualify as a drug candidate, a compound should be able to get to its biological target at an effective concentration and be able to stay in the body long enough to elicit the desired therapeutic effect^52^. The in-silico ADMET analysis was done to determine these properties in which ADMET is described as absorption, distribution, metabolism, excretion, and toxicity. These pharmacokinetic and safety parameters are an essential aspect of drug discovery in early stages because a significant percentage of the drug candidates fail in the drug development process owing to unfavorable ADMET profiles^53^. The web-based tool pkCSM was used to predict the ADMET properties of the chosen emodin-based compounds in the present study by pkCSM(https://biosig.lab.uq.edu.au/pkcsm/prediction). The PubChem database was used to retrieve canonical SMILES of all compounds, which were then used as input to both platforms^54^. These tools use validated computational models to predict important pharmacokinetic and toxicity parameters, such that early compounds that possess desirable drug-like and safety properties are readily identified, and compounds with unfavorable profiles are excluded from further structure-based and dynamic analyses^55^.

### MD simulations

Molecular dynamics (MD) simulations were carried out on selected PARP-1-ligand complexes to assess their stability and dynamic behavior under near-physiological conditions using the GROMACS simulation package^20^. The docked protein–ligand complexes were placed in an orthorhombic simulation box with dimensions (approximately 8.72 × 7.03 × 7.73 nm3), providing sufficient space for atom movements. The box was filled with explicit water molecules to solvate the system. NaCl was added to the box to neutralize the simulation systems and to achieve sufficiently high ionic strength (0.15 M)^56,57^.

Before running the simulation, all systems were initially energy minimized to remove steric clashes and poor contacts within the system. The systems were individually equilibrated under constant volume, and temperature (NVT) as well as constant pressure and temperature (NPT) conditions^58^. During the equilibration, the temperature was maintained at 300 K using the v-rescale thermostat while the pressure was maintained at 1 bar using pressure coupling with separate coupling groups used for the protein, ligand, and solvent/ions^59,60^. After the equilibration processes, a 500 ns production MD simulation was performed under NPT conditions using the Parrinello-Rahman barostat. The long-range electrostatic interactions were treated by the Particle Mesh Ewald (PME) method, whereas short-range Coulomb and van der Waals interactions were calculated with a cutoff distance of 1.2 nm^61^. All covalent bonds, including hydrogen, involved in the system were constrained using the LINCS algorithm, allowing an integration time step of 2 fs^62^. Coordinates, energies, and the trajectory of the system were saved with a time interval of 2 ps for further analysis, including RMSD, RMSF, Rg, SASA, MolSA, hydrogen-bonding, PCA, and DCCM^63,64^.

## | Result and discussion

### PASS-based QSAR analysis

The profile of biological activity of the chosen phytochemicals was predicted by the use of the PASS analysis, and the obtained probability of activity (Pa) and inactivity (Pi) values are presented in Table 2. In general, most of the compounds had high Pa and low Pi values, which was a clear indication of high chances of biological relevance. To achieve antineoplastic activity, most of the ligands contained Pa values greater than 0.70, and compounds including L01, L03, L04, L08, L09, L11, and L14 exhibited a high predicted activity (Pa 0.77- 0.84) and a low Pi value (< 0.02). Equally, the antimutagenic potential was competitive throughout the set of ligands, with Pa values often surpassing 0.80, indicating an advantageous contribution to mitigating cellular damage that is attributed to mutation. Moreover, the compounds also had significant histidine kinase inhibition and reductant activities, with some such ligands (e.g., L06, L08, and L11) having a Pa value of above 0.85 in histidine kinase inhibition and up to 0.90 in reductant activity. Such activities are applicable within the framework of cancer signaling and control of oxidative stress. Combinations of the PASS prediction spectrum indicate that most of the study phytochemicals have highly active anti-cancer profiles, which allows their application to additional structure-based docking and molecular dynamics studies. This finding is important because it supports the initial hypothesis that emodin-derived anthraquinones are not random chemical candidates but biologically relevant scaffolds with predicted cancer-related activity. Therefore, PASS analysis provided the first level of evidence that these compounds could be rationally explored as alternative PARP-1 inhibitor candidates.

**Table 2:** PASS-predicted biological activity profiles of emodin-based anthraquinone analogues. The table summarizes the predicted probability of activity (Pa) and probability of inactivity (Pi) for anticancer-related biological activities, including antineoplastic, antiseptic, histidine kinase inhibitory, reductant, and antimutagenic effects. Higher Pa values and lower Pi values indicate a greater predicted likelihood that the compound may show the corresponding biological activity.

PubChem ID	Assigned ligand no	Parameter of biological PASS prediction									
		Antineoplastic		Antiseptic		Histidine kinase inhibitor		Reductant		Antimutagenic	
		Pa	Pi	Pa	Pi	Pa	Pi	Pa	Pi	Pa	Pi
10086148	L01	0.773	0.015	0.857	0.004	0.856	0.003	0.804	0.004	0.839	0.003
10207	L02	0.707	0.025	0.878	0.003	0.809	0.004	0.662	0.008	0.760	0.005
10425624	L03	0.781	0.014	0.840	0.004	0.764	0.005	0.804	0.004	0.569	0.011
10469084	L04	0.722	0.022	0.778	0.004	0.850	0.003	0.603	0.011	0.535	0.013
10639	L05	0.802	0.012	0.179	0.081	0.714	0.007	0.326	0.075	0.466	0.017
122635	L06	0.840	0.008	0.201	0.072	0.761	0.005	0.656	0.008	0.408	0.022
122840	L07	0.773	0.015	0.857	0.004	0.856	0.003	0.804	0.004	0.839	0.003
12313148	L08	0.773	0.015	0.857	0.004	0.856	0.003	0.804	0.004	0.839	0.003
160717	L09	0.763	0.017	0.835	0.004	0.846	0.003	0.784	0.004	0.840	0.003
318730	L10	0.601	0.045	0.663	0.006	0.792	0.004	0.641	0.009	0.820	0.004
3220	L11	0.791	0.013	0.887	0.003	0.902	0.002	0.872	0.003	0.860	0.003
343129	L12	0.692	0.028	0.689	0.005	0.864	0.003	0.903	0.003	0.799	0.004
361515	L13	0.759	0.017	0.829	0.004	0.836	0.003	0.778	0.004	0.818	0.004
626899	L14	0.766	0.014	0.829	0.004	0.836	0.003	0.778	0.004	0.818	0.004
9866696	L15	0.811	0.010	0.825	0.004	0.856	0.003	0.743	0.005	0.876	0.003

### Drug-likeliness

Table 3 summarizes the physicochemical and drug-likeness properties of the chosen ligands and suggests a generally good structure-based screening profile. The molecular weights of most compounds are moderate, and in the case of small-molecule binding, the range is 256–312 g/mol. The ligands have a low number of rotatable bonds (0–3), and this implies that the ligands do not possess much conformational flexibility, and they tend to establish stable interactions with the binding pocket of the protein. The hydrogen-bonding capacity of the set of ligands is also balanced, and most compounds have between 4–6 hydrogen-bond acceptors and 2–3 hydrogen-bond donors, which are useful in polar interactions with active-site residues.

**Table 3:** Physicochemical and drug-likeness properties of the selected emodin-based anthraquinone analogues. The table summarizes key molecular descriptors, including molecular weight, rotatable bonds, hydrogen-bond acceptors and donors, molar refractivity, topological polar surface area (TPSA), lipophilicity, aqueous solubility, gastrointestinal absorption, blood–brain barrier permeability, Lipinski rule compliance, bioavailability score, and synthetic accessibility. These parameters were used to evaluate whether the selected ligands possess suitable lead-like and oral drug-like properties before molecular docking and molecular dynamics simulation studies.

Ligand	Molecular weight(g/mol)	Num. rotatable bonds	Hydrogen bond acceptor	Hydrogen bond donor	Molar refractivity	TPSA, A2	Consensus Log Po/w	Log S (ESOL)	G.I. absorption	BBB permeant	Lipiniski rule		Bioavailability score	Synthetic accessibility
											Result	Violation		
L01	300.26	1	6	3	77	104	1.82	− 3.73	High	No	Yes	0	0.55	2.72
L02	270.24	1	5	3	70	95	1.5	− 3.04	High	No	Yes	0	0.55	2.6
L03	312.27	3	6	2	80	101	1.94	− 3.48	High	No	Yes	0	0.55	2.85
L04	284.26	1	5	2	75	84	2.19	− 3.87	High	No	Yes	0	0.55	2.66
L05	284.26	1	5	2	75	84	2.27	− 3.87	High	No	Yes	0	0.55	2.69
L06	256.25	0	4	3	70	78	2.37	− 3.92	High	No	Yes	0	0.55	2.55
L07	256.25	1	4	3	70	78	1.96	− 3.29	High	No	Yes	0	0.55	2.58
L08	300.26	1	6	3	77	104	1.82	− 3.73	High	No	Yes	0	0.55	2.72
L09	284.26	1	5	2	75	84	2.2	− 3.87	High	No	Yes	0	0.55	2.68
L10	432.4	3	10	6	103	174	0.1	− 3.18	Low	No	Yes	1	0.55	5.06
L11	270.24	0	5	3	71	95	1.87	− 3.67	High	No	Yes	0	0.55	2.57
L12	396.3	6	8	0	99	113	2.72	− 3.78	High	No	Yes	0	0.55	3.26
L13	298.29	2	5	1	80	73	2.6	− 4.08	High	Yes	Yes	0	0.55	2.79
L14	298.29	2	5	1	80	73	2.61	− 4.08	High	Yes	Yes	0	0.55	2.79
L15	418.39	3	9	7	102	168	− 0.07	− 2.46	Low	No	Yes	1	0.55	4.97

The parameters of polarity and lipophilicity also facilitate the appropriateness of these molecules for docking. TPSA values of most ligands are less than 120 Å^2^ and consensus logP values range from 1.5 to 2.7, which reflect a good balance of hydrophilicity and hydrophobicity. The ESOL logS values are predicted to be within an acceptable range, and it is expected that most compounds will have high gastrointestinal absorption, implying good drug-like properties^64^. Conversely, compounds L10 and L15 have a relatively higher molecular weight, polarity, lower predicted GI absorption, and one violation of Lipinski^64^. However, these molecules are kept in a reasonable chemical space and were not eliminated in order to prevent the elimination of potentially active scaffolds at this point^65^.

Notably, all ligands comply with or exhibit little deviation from the Lipinski Rule of Five, and all have the same bioavailability score of 0.55, suggesting similar oral drug-likeness. Based on the physicochemical properties and the drug-likeness assessment of the considered anthraquinone derivatives, most of the studied compounds are placed within an acceptable lead-like chemical space. Importantly, the predicted PARP-1 binding potential of the studied ligands is not related to the introduction of unfavorable basic physicochemical properties. Thus, the selected ligands are considered suitable for the assessment by means of molecular docking and molecular dynamics simulations. These studies will provide insight into the binding of the studied compounds as well as into their stability and into their ability to act as PARP-1 inhibitors.

### In-silico ADME evaluation

An in silico ADME assessment (Absorption, Distribution, Metabolism, and Excretion) of the docked PARP-1 inhibitors was conducted using the Deep-PK and pkCSM prediction platforms to further examine the drug-likeness and clinical feasibility of the docked inhibitors^66–68^. Table 4 shows the predicted pharmacokinetic values of the chosen ligands and were evaluated to select compounds with good oral drug-like properties. Regarding absorption, the majority of ligands showed favorable predicted Caco-2 cell permeability values. The compounds with predicted log Papp values greater than approximately -5.00 are generally considered to have moderate to high intestinal epithelial uptake and oral absorption^31,69^. Additionally, most of the compounds were estimated to be strongly absorbed in the human intestine and to have acceptable human oral bioavailability at 20% and 50% concentrations. Conversely, L10 and L15 ligands exhibited comparatively low predicted Caco-2 permeability and were classified as non-absorbable, indicating low potential for oral absorption potential compared to the other ligands^69,70^. The parameters of distribution also showed that the majority of ligands were predicted to be non-penetrant to the blood–brain barrier (BBB), which is preferable when central nervous system-related adverse effects are minimized in non-neurological indications^71^. The proportion of unbound drug in human plasma (fu,p) was moderate to high in the entire data set, showing that a significant level of the given drug would be pharmacologically active, and the plasma protein binding would be balanced^72^. In addition, the projected constant volume of distribution (log VDss) data indicated moderate to high tissue distribution of most compounds, which indicated effective systemic exposure^73^. The metabolic profiling revealed that several ligands could be used as inhibitors of major cytochrome P450 (CYP450) isoenzymes, with CYP1A2 and CYP2C19 as the most common, and with CYP2D6 and CYP3A4 having fewer, respectively^10,13,74,75^. This trend indicates that there is a controllable risk of metabolic drug-drug interactions for the chosen candidates. Excretion profile revealed that the clearance of most of the ligands was moderate, and most importantly, none of them was predicted to block the renal Organic Cation Transporter-2 (OCT2), suggesting that transporter-mediated renal toxicity was unlikely to occur^76,77^. Moreover, the predicted half-life of all ligands was short (less than 3 h), potentially resulting in the possibility of a flexible dosing schedule and decreasing the probability of long-term accumulation^78^. All in all, incorporation of the ADME parameters makes L01, L04, L08, L09, and L13 the most promising oral drugs. This group of ligands is characterized by good intestinal absorption, good oral bioavailability, low BBB penetration, balanced plasma protein binding, acceptable metabolism, and acceptable clearance properties. The compounds were, therefore, deemed to be the best to do additional molecular dynamics simulations and experimental validation to determine their stability and effectiveness at physiological conditions^55,79^. These ADME findings are directly relevant to the study objective because a potential PARP-1 inhibitor must show not only strong target binding but also acceptable pharmacokinetic behavior. The favorable absorption, bioavailability, limited BBB penetration, and acceptable clearance profiles observed for several ligands indicate that these compounds may have practical potential for further optimization as orally available anticancer lead candidates.

**Table 4:** In silico ADME and pharmacokinetic profiles of selected emodin-based PARP-1 inhibitor candidates. The table summarizes predicted absorption, distribution, metabolism, and excretion parameters, including Caco-2 permeability, intestinal absorption, oral bioavailability, BBB permeability, plasma protein binding, volume of distribution, CYP inhibition, clearance, half-life, and OCT2 inhibition. These properties were used to assess the pharmacokinetic suitability of the ligands before further docking and molecular dynamics evaluation.

Ligand	Assigned ligand no	Absorption				Distribution				Metabolism					Excretion		
		Caco-2 (logPaap)	Human intestinal absorption (AB/NAB)	Human Oral bioavailability 20% (BA/NBA)	Human oral bioavailability 50% (BA/NBA)	Blood–brain barrier (P/NP)	Fraction unbound (human)	Plasma protein binding	Steady state volume of distribution (log VDss)	CYP 1A2 (I/NI)	CYP 2C19 (I/NI)	CYP 2C9 (I/NI)	CYP 2D6 (I/NI)	CYP 3A4 (I/NI)	Clearance log (ml/min/kg)	Half-Life of drug	Organic cation Transporter 2 (I/NI)
10086148	L01	− 4.88	A	B	B	NP	0.94	69.05	0.6	I	I	NI	I	I	9.31	< 3hs	I
10207	L02	− 4.8	A	B	B	P	0.76	58.72	0.83	I	I	NI	I	NI	7.29	< 3hs	NI
10425624	L03	− 4.61	A	NB	B	P	0.89	54.96	0.9	I	I	I	I	I	7.94	< 3hs	NI
10469084	L04	− 4.47	A	B	B	P	0.97	64.2	0.61	I	I	NI	I	I	5.82	< 3hs	NI
10639	L05	− 4.43	A	B	B	P	0.93	58.4	0.7	I	I	I	I	I	6.75	< 3hs	NI
122635	L06	− 4.8	A	B	B	P	0.73	60.8	0.78	I	I	I	I	I	6.61	< 3hs	NI
122840	L07	− 4.85	A	B	B	P	0.6	53.6	0.9	I	I	I	I	NI	6.02	< 3hs	NI
12313148	L08	− 4.89	A	B	B	NP	0.86	69.3	0.47	I	I	NI	I	NI	8.8	< 3hs	NI
160717	L09	− 4.45	A	B	B	NP	0.92	59.67	0.71	I	I	NI	I	I	7.13	< 3hs	NI
318730	L10	− 6.88	NA	NB	NB	NP	0.78	22.01	1.03	I	NI	NI	NI	NI	11.32	< 3hs	NI
3220	L11	− 4.87	A	B	B	NP	0.83	65.26	0.61	I	I	NI	I	I	7.28	< 3hs	NI
343129	L12	− 4.47	A	NB	B	P	0.42	40.94	1.79	I	NI	NI	NI	NI	4.86	< 3hs	NI
361515	L13	− 4.49	A	B	B	P	0.99	57.2	0.64	I	I	NI	I	I	5.62	< 3hs	NI
626899	L14	− 4.57	A	B	B	P	0.86	54.27	0.9	I	I	NI	I	I	6.67	< 3hs	NI
9866696	L15	− 6.41	NA	NB	NB	NP	0.7	68.66	1.27	NI	NI	NI	NI	NI	11.63	< 3hs	NI

BA = Bioavailable, NBA = Non-Bioavailable; I = Inhibitor, NI = Non-Inhibitor; AB = Absorbed, NAB = Non-Absorbed; P = permeant; NP = non-permeant; BBB = blood–brain barrier; OCT2 = organic cation transporter 2.

### Aquatic and non-aquatic toxicity profile

Tables 5 and 6 summarize the predicted aquatic and non-aquatic toxicity profiles of the selected ligands using in-silico models. Overall, most compounds displayed favorable environmental and systemic safety features, with consistent predictions of non-carcinogenicity and good biodegradability across the ligand set. Bioconcentration factor (BCF) values were mostly in low-to-moderate range (log10 L/kg), indicating negligible bioaccumulation potential in aquatic ecosystems, and only a few ligands (e.g., L10 and L15) exhibited particularly low BCF values, corresponding to rapid environmental clearing^8,9,80,81^. Despite most compounds being classified into the toxic towards crustacean’s category, both predicted Daphnia magna and fathead minnow acute toxicity values were in the range generally found for aromatic polyphenolic scaffolds, reflecting a manageable aquatic risk profile at environmentally relevant concentrations. In the non-aquatic toxicity assessment, the ligands were mostly predicted to be non-mutagenic in the AMES test, non-corrosive to the eye, and non-inhibitory towards the hERG I channel, implying a low risk of genotoxicity, ocular toxicity, and cardiotoxic liability. However, several compounds showed predicted liver injury (DILI) alerts, which are a recognized shortcoming of anthraquinone scaffolds and warrant focused evaluation of hepatotoxicity in further experimental follow-up studies^8–10,82,83^. Importantly, the predicted maximum tolerated dose (MTD), oral rat acute toxicity (LD_50_), and chronic toxicity (LOAEL) values provide acceptable systemic exposure margins for most ligands, which are taken as evidence for their suitability as orally administered drug candidates^8,9,13,81,84^. Collectively, these results suggest that the prioritized compounds, especially L03 (CID: 10425624), L08 (CID: 12313148), and L13 (CID: 361515), have an overall balanced predicted toxicity profile, with no major genotoxic, cardiotoxic, ocular toxicity, or renal transporter-related liabilities. Although the predicted liver injury alerts indicate that hepatotoxicity should be carefully examined in future experimental studies, the overall safety profile supports the continued evaluation of these compounds as early-stage PARP-1 inhibitor leads.

**Table 5:** Predicted aquatic toxicity profiles of selected emodin-based anthraquinone ligands. The table presents silico environmental toxicity indicators, including bioconcentration factor, biodegradation, carcinogenicity, crustacean toxicity, and predicted acute toxicity toward Daphnia magna and fathead minnow. These parameters were used to estimate the potential aquatic safety and environmental risk of the selected ligands.

CID	Aquatic toxicity						
	Ligand	Bioconcentration factor log10(L/kg)	Biodegradation (T/S)	Carcinogenesis (T/S)	Crustacean (T/S)	aphnia Magna − log10[(mg/L)/(1000*MW)]	Fathead Minnow − log10[(mg/L)/(1000*MW)]
10086148	L01	1.01	Safe	Safe	Toxic	4.95	4.25
10207	L02	0.83	Safe	Safe	Toxic	3.83	4.1
10425624	L03	1.13	Safe	Safe	Toxic	5.73	4.45
10469084	L04	1.42	Safe	Safe	Toxic	4.5	4.42
10639	L05	1.39	Safe	Safe	Toxic	5.34	4.44
122635	L06	1.33	Safe	Safe	Toxic	4.56	4.2
122840	L07	1.05	Safe	Safe	Toxic	3.91	4.12
12313148	L08	0.64	Safe	Safe	Toxic	4.91	4.13
160717	L09	1.31	Safe	Safe	Toxic	5.45	4.45
318730	L10	− 1.26	Safe	Safe	Safe	4.88	4.22
3220	L11	1.05	Safe	Safe	Toxic	4.6	4.12
343129	L12	1.05	Safe	Safe	Toxic	6.87	5.15
361515	L13	1.81	Safe	Safe	Toxic	5.82	4.7
626899	L14	1.6	Safe	Safe	Toxic	6.29	4.66
9866696	L15	− 2.08	Safe	Safe	Safe	4.84	3.81

T = toxic; S = safe; MW = molecular weight.

**Table 6:** Predicted non-aquatic toxicity profiles of selected emodin-based anthraquinone ligands. This table reports in silico toxicity endpoints, including AMES mutagenicity, eye corrosion, hERG I inhibition, drug-induced liver injury, maximum tolerated dose, oral rat acute and chronic toxicity, respiratory toxicity, and skin sensitization. These parameters were used to evaluate possible systemic safety liabilities of the selected PARP-1 inhibitor candidates.

Non-aquatic toxicity											
CID	Ligand	AMES mutagenesis (T/S)	Eye Corrosion	hERG I inhibitor (T/S)	Liver injury I (DILI)	Liver injury II	Maximum tolerated dose (log mg/kg/day)	Oral rat acute Tity (LD50) (mol/kg)	Oral rat chronic Tity (LOAEL) (log mg/kg_bw/day)	Respiratory disease (T/S)	Skin sensitisation (T/S)
10086148	L01	Toxic	Safe	Safe	Toxic	Toxic	0.84	2.46	3.26	Safe	Safe
10207	L02	Toxic	Safe	Safe	Toxic	Toxic	0.99	2.45	3.4	Safe	Safe
10425624	L03	Toxic	Safe	Safe	Toxic	Toxic	1.2	2.66	2.92	Safe	Safe
10469084	L04	Safe	Safe	Safe	Toxic	Toxic	1.12	2.36	3.02	Safe	Safe
10639	L05	Toxic	Safe	Safe	Toxic	Toxic	1.18	2.4	2.94	Safe	Safe
122635	L06	Safe	Safe	Safe	Toxic	Toxic	0.87	2.29	3.05	Safe	Toxic
122840	L07	Toxic	Safe	Safe	Toxic	Toxic	0.75	2.26	3.18	Toxic	Toxic
12313148	L08	Toxic	Safe	Safe	Toxic	Toxic	1.17	2.55	3.3	Toxic	Toxic
160717	L09	Safe	Safe	Safe	Toxic	Toxic	1.04	2.36	2.95	Safe	Safe
318730	L10	Toxic	Safe	Safe	Safe	Toxic	0.66	2.5	4.22	Safe	Safe
3220	L11	Safe	Safe	Safe	Toxic	Toxic	1.03	2.47	3.32	Safe	Toxic
343129	L12	Safe	Safe	Safe	Toxic	Toxic	1.71	2.47	2.61	Safe	Safe
361515	L13	Safe	Safe	Safe	Toxic	Toxic	1.36	2.35	2.68	Safe	Safe
626899	L14	Safe	Safe	Safe	Toxic	Toxic	1.1	2.32	2.59	Safe	Safe
9866696	L15	Toxic	Safe	Safe	Safe	Toxic	0.72	2.63	4.28	Toxic	Safe

T = toxic; S = safe; DILI = drug-induced liver injury; hERG = human ether-à-go-go-related gene; LD50 = median lethal dose; LOAEL = lowest observed adverse effect level.

### Molecular docking and binding affinity analysis

To determine the binding capacity of the identified ligands to the catalytic site of PARP-1 (PDB ID: 7KK4), molecular docking was used. AutoDock Vina was used to run docking simulations and the binding free energies (kcal/mol) were determined^49^. The docking scores of all screened ligands are summarized in Supporting Information Table S1, and the best complexes were chosen to proceed to interaction analysis.

The binding affinity was high for all the ligands studied, with docking scores of about 8.9–10.8 kcal/mol, showing energetically favorable interactions in the PARP-1 active site^85^. The most active of them were Lig03 (CID: 10425624), Lig10 (CID: 318730), Lig15 (CID: 9866696), and STD (CID: 24958200), which showed the highest binding affinities. It is important to note that Lig10 exhibited the best predicted binding affinity -10.8 (kcal/mol), indicating a highly stable protein–ligand complex. These top-ranked ligands were chosen for further dynamic interaction analysis with the target protein based on the docking scores and consistency of binding poses^86^. The docking results are significant because they indicate that several emodin-derived anthraquinones can occupy the PARP-1 catalytic pocket with predicted affinities comparable to or stronger than the reference inhibitor. This supports the central objective of the study by showing that the anthraquinone scaffold may provide a structurally distinct starting point for PARP-1 inhibitor development.

### Protein–ligand interaction and active site analysis

**Fig. 2:**
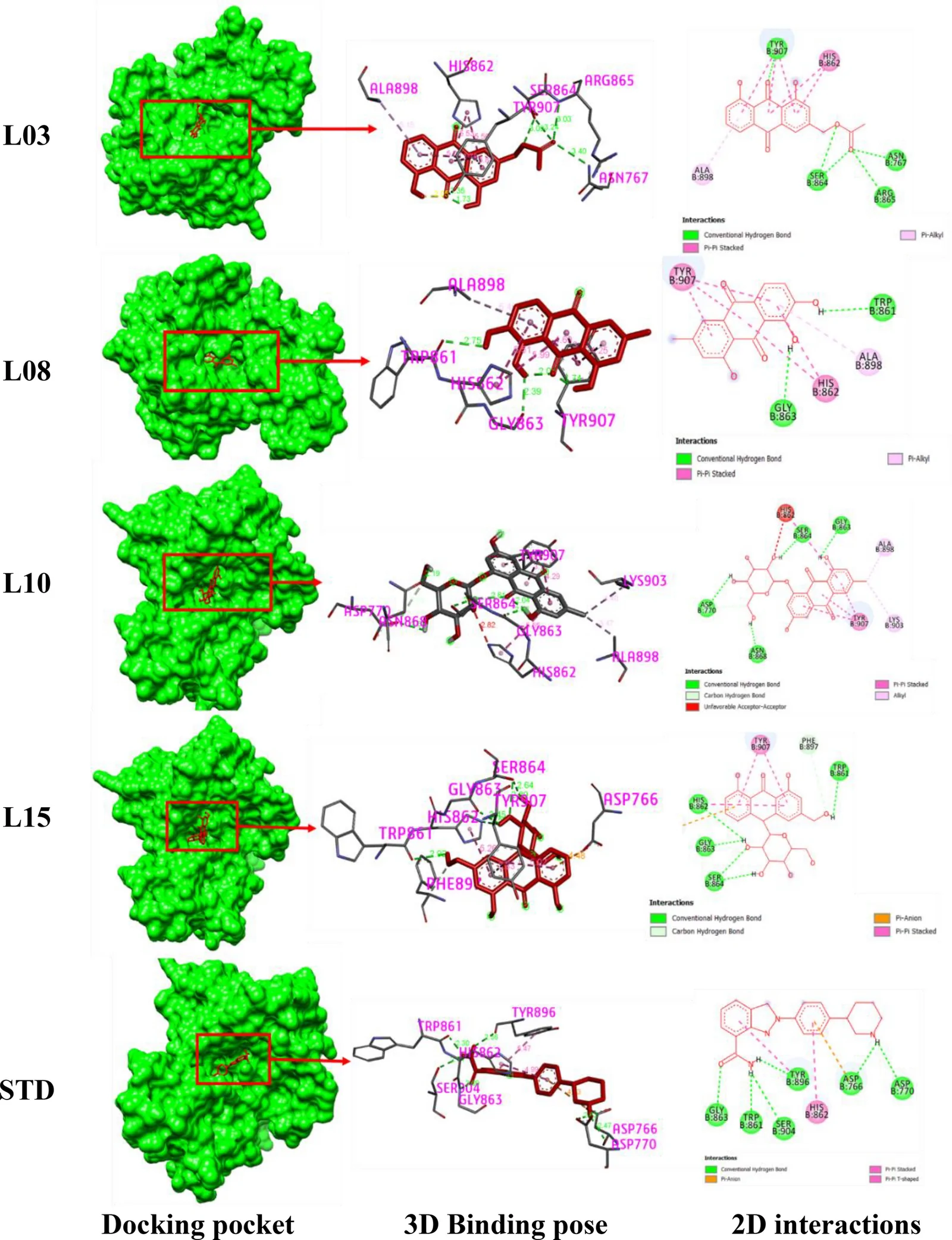
Protein–ligand interaction profiles of the selected ligands with the catalytic site of PARP-1. Surface view of PARP-1 highlighting the NAD^+^ binding pocket (left), three-dimensional docked pose (middle), and two-dimensional interaction showing key hydrogen binding and π-interactions with the active-site residues.

In order to study the molecular mechanisms of ligand recognition, the interaction analysis of the best ligand complexes of PARP-1 was conducted in detail using BIOVIA Discovery Studio Visualizer. Interaction profiles, such as interaction type, residue contacts, and bond-distance ranges, are presented in Table 7 and Fig. 2, whereas the examples of binding modes are presented in Fig. 2. The binding affinity of the Protein-Lig03 complex (CID: 10425624) was -10.0 kcal/mol, and it formed several stabilizing interactions with several important residues of PARP-1, such as ALA898, HIS862, SER865, ARG865, TYR907, and ASN767. Ten interactions were found, which included carbon-hydrogen bonds, p-p stacked interactions, p-p T-shaped interactions, p-alkyl contacts, and the bond distance was between 1.73 and 5.56 Å. Similarly, the most favorable docking score, at -10.8 kcal/mol, was the Protein-Lig10 complex (CID: 318730), which interacted with a wide range of amino acid residues ASP770, ASN868, SER864, TYR907, GLY863, HIS862, and LYS903, and these eleven interactions were dominated by carbon-hydrogen bonds and π–π stacking. The protein-Lig15 complex (CID: 9866696) exhibited a binding affinity of -10.0 kcal/mol and formed 9 interactions with the following residues: TRP861, PHE897, HIS862, GLY863, SER864, TYR907, and ASP766. The π–π stacked and π-anion interactions further stabilized this complex, thus showing a strong contribution by electrostatic and aromatic forces. Comparatively, the standard compound (STD, CID: 24958200) had nine interactions with the residues TRP861, TYR896, HIS862, SER904, GLY863, ASP766, and ASP770 and a binding affinity of -9.9 kcal/mol. The interaction profile of the standard ligand was stable, but several investigational ligands showed the same or better binding affinity and interaction density, indicating a greater stabilization in the PARP-1 catalytic pocket. Altogether, the analysis of interaction indicated that the chosen ligands bind to conserved residues of the NAD+-binding region of PARP-1 mostly by incorporating a mixture of hydrogen bonding, π–π stacking, and hydrophobic interactions. The identified ligands having better binding scores compared to the standard drug were chosen to undergo further molecular dynamics simulation to determine their stability under physiological conditions^41^. In addition to the top-scoring ligands, one molecule with a slightly lower docking score than the standard drug was included intentionally to examine whether favorable dynamic behavior could offset a lower docking affinity^87^. The interaction analysis further explains why these ligands may be relevant for PARP-1 inhibition: several candidates contacted conserved residues within the NAD+-binding region through hydrogen bonding, π–π stacking, and hydrophobic interactions. Such interactions suggest that these compounds may interfere with catalytic-site recognition in a manner similar to established PARP-1 inhibitors, while retaining a distinct anthraquinone-based chemical framework.

**Table 7:** Docking interaction profiles of the top-scoring emodin-based anthraquinone analogues and the standard compound with PARP-1. This table reports the predicted binding affinity, interacting catalytic-site residues, interaction types, number of contacts, and bond-distance ranges for each protein–ligand complex. These interaction profiles were used to compare how the selected ligands bind within the PARP-1 active site relative to the standard compound.

Complex	Binding affinity (Kcal/mol)	Residue in contact	Interaction number and type	Bond distance range (Å)
Protein-Lig03 (10425624) complex	− 10.0	ALA898, HIS862, SER865, ARG865, TYR907, ASN767	11(5-CHB, 4-Pi-Pi Stacked, 1 Pi-Pi T-shaped, 1 Pi-Alkyl)	1.73–5.56
Protein-Lig08 (12313148) complex	− 9.5	ALA898, TRP861, HIS862, GLY863, TYR907	7 (2-CHB, 4-Pi-Pi Stacked,1-Pi-Alkyl)	1.74–5.99
Protein-Lig10 (318730) complex	− 10.8	ASP770, ASN868, SER864, TYR907, GLY863, HIS862, LYS903, ALA898	11 (4-CHB, 4-Pi-Pi Stacked, 1-Pi-Alkyl, 2-Pi-Alkyl)	2.04–5.65
Protein-Lig15 (9866696) complex	− 10.0	TRP861, PHE897, HIS862, GLY863, SER864, TYR907, ASP766	9 (5-CHB, 3-Pi-Pi Stacked, 1-Pi-Anion)	2.45–5.62
Protein-Std (24958200) complex	− 9.9	TRP861, TYR896, HIS862, SER904, GLY863, ASP766, ASP770	9 (6-CHB, 1-Pi-Pi Stacked, 1-Pi-Pi T-shaped, 1-Pi-Anion)	2.30–5.47

CHB = carbon-hydrogen bond; Pi-Pi = π–π interaction; Pi-Alkyl = π–alkyl interaction; Pi-Anion = π–anion interaction; Std = standard compound.

### Molecular dynamics simulations

Molecular dynamics (MD) simulations used in structure-based drug design can provide a more in-depth understanding of the structural stability, conformational behavior, and intermolecular forces of protein–ligand complexes compared with results of docking. A 500 ns MD simulation was conducted in this study to determine the stability of the chosen complexes under dynamic settings under near-physiological conditions^8,9,88,89^. Various metrics were used for post-simulation trajectory analysis including protein and ligand root mean square deviation (RMSD) to measure overall stability, root mean square fluctuation (RMSF) to measure residue flexibility, and principal component analysis (PCA) to describe the predominant collective motions of the protein during the simulation^38,90–92^. Moreover, the persistence and strength of the intermolecular interactions between the protein and ligands of interest were investigated using hydrogen bond analysis^92^. The analysis of changes in structural compactness and solvent exposure was performed using molecular surface area (MolSA) and solvent-accessible surface area (SASA)^93,94^. The potential energy and interaction energy profiles calculated during the simulation were also used to determine the energetic stability of the complexes. A combination of these parameters allows a full understanding of conformational stability, consistency of binding, and dynamic behavior of the protein–ligand systems. The overall findings of the RMSD, RMSF, PCA, hydrogen bonding, MolSA, SASA, and energy analyses provide a combined assessment of the dynamic behavior of the studied complexes. This analysis is important because docking alone cannot confirm whether a ligand remains stable in the binding pocket under time-dependent physiological motion. Therefore, the MD simulations were used to determine whether the initially favorable docking poses translated into persistent binding, structural stabilization, and coordinated protein dynamics, which are essential features for prioritizing realistic PARP-1 inhibitor candidates.

#### Analysis of protein RMSD

**Fig. 3:**
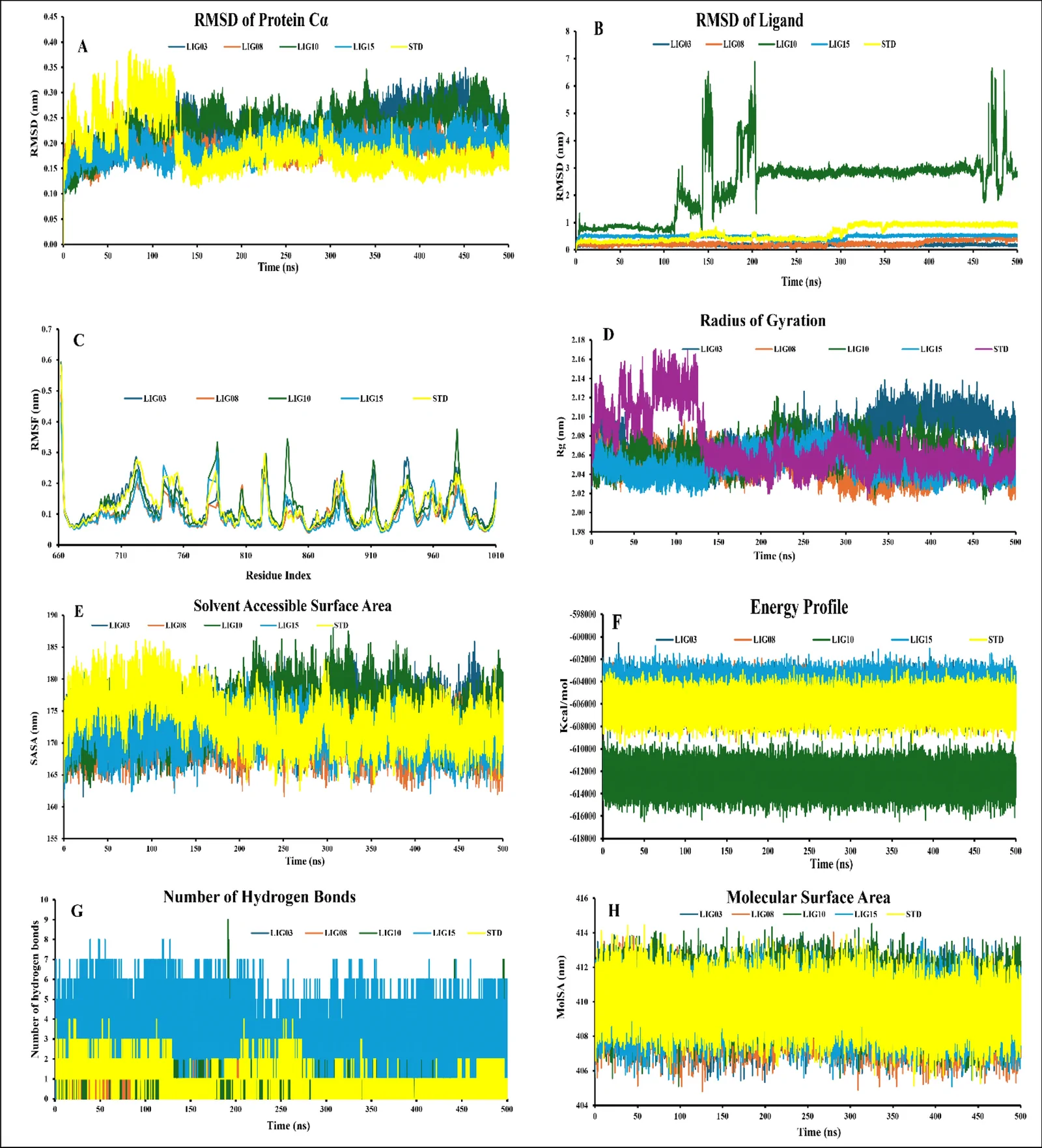
Comparative molecular dynamics (MD) analyses of the PARP-1–ligand complexes. Time-dependent profiles of key structural and energetic parameters—(**A**) Protein Cα RMSD; (**B**) Ligand RMSD; (**C**) RMSF; (**D**) Radius of Gyration (Rg); (**E**) Solvent-Accessible Surface Area (SASA); (**F**) Energy Profile; (**G**) number of hydrogen bonds, and (**H**) Molecular Surface Area (MolSA). These parameters were used to compare structural stability, residue flexibility, compactness, solvent exposure, energetic stability, and interaction persistence among the selected ligands and the standard compound.

Root mean square deviation (RMSD) is a commonly used structural measure for the description of the conformational stability of protein structure that is often used to examine the stability of changes in protein structure during molecular dynamics simulations by determining the average change in the position of backbone atoms^38,91,95^. In the current work, the 500 ns long molecular dynamics were carried out for the protein complexes bound to the chosen ligands (L03, L08, L10, L15) and the standard compound, and the RMSD profile of the backbone was compared (Fig. 3A). In general, the RMSD trajectories of all protein–ligand complexes remained steady over the entire simulation duration with slight variations, which is a sign of well-equilibrated systems. The average RMSD of the protein backbone was also in the range of 0.189–0.223 nm, which is deemed acceptable to ensure structural integrity during MD simulations. The complexes with L08 and L15 exhibited a relatively smaller mean RMSD, which indicates that the protein structure was more stabilized, but the standard compound had a little higher deviation. The highest RMSDs recorded in the course of the simulations did not surpass about 0.35 nm in the case of the ligand bound complexes, and the standard complex had slightly higher peak deviation, which indicates a relatively higher flexibility. There were fluctuating results at the beginning of the simulation, which was the equilibration of the system, and then all the complexes came to rest, and had fixed conformations without any sudden structural changes. Collectively, the RMSD analysis indicates that all the chosen ligands can create structurally stable protein–ligand complexes, and some of the candidates were equally or more stable than the standard compound. Such results allow a conclusion about the validity of the MD simulations and the importance of conducting further dynamic and energetic studies of the chosen complexes. From a biological perspective, the stable backbone RMSD values suggest that ligand binding did not destabilize the PARP-1 catalytic domain. This is an important requirement for lead selection because a promising inhibitor should maintain a stable complex with the target protein rather than causing large structural disruption or rapid conformational drift.

#### Analysis of ligand RMSD

To measure the stability in the positional and conformational integrity of the ligands in the protein binding pocket throughout the 500 ns molecular dynamics simulation, ligand root mean square deviation (RMSD) analysis was conducted^91,96^. Ligand RMSD gives an understanding of the ability of a compound to retain its docked orientation under dynamic conditions and a direct measure of binding strength. The direct comparison between the RMSD profiles of the selected ligands and the standard compound (CID: 24958200) is shown in Fig. 3B. The compounds were tested and the one, L03 (CID: 10425624), with a mean ligand RMSD of 0.182 nm had a range of 0.0005 to 0.306 nm, indicating high positional stability in the binding site. The average RMSD of the standard compound was higher than that of L03 (0.604 nm vs. 0.182 nm), and the maximum deviation was also higher (1.061 nm vs. 0.306 nm), suggesting that the overall stability of the L03 ligand was higher than that of the standard drug. This implies that L03 is like the standard ligand in terms of stability and has slightly reduced peak deviations. L08 showed relatively low RMSD, with a mean value of 0.234 nm and a maximum deviation of 0.571 nm, suggesting moderate fluctuations while staying in the binding pocket. In comparison, L10 (CID: 318730) was much larger with mean RMSD of 2.389 nm with a maximum deviation of 6.886 nm and no binding stability and strong conformational motion. Such enormous deviations suggest that L10 is in the binding pocket significantly rearranged in its position, and therefore dynamically incompetent to the normal ligand. Similarly, the mean RMSD of L15 (CID: 9866696) was 0.482 nm and maximum was 0.647 nm which is much larger in comparison with the standard compound. Although L15 was not discharged in the simulation, when it was occupied in the active site it reflected higher RMSD values that could be attributed to enhanced flexibility and low conformational restraint that means that it is not as dynamically stable as the norm. The overall result of the ligand RMSD analysis is that the dynamic behavior of the compounds in tests is clearly different compared to that of the standard drug. L03 (CID: 10425624) seems to be the most suitable one that has the same stability as the standard compound but is slightly more stable, whereas L10 and L15 are highly variable with low stability. These data are a solid clue that L03 is superior or equivalent candidate to the standard drug in terms of ligand-level dynamic stability and why it should be prioritized among other candidates that are to be further confirmed through other mechanistic and experimental means. This result implies that docking affinity alone was not sufficient for final candidate ranking. Although L10 showed strong docking affinity, its high ligand RMSD suggests poor positional stability, whereas L03 maintained its binding orientation more effectively over 500 ns. Therefore, L03 was prioritized because it combined favorable docking with superior dynamic retention in the PARP-1 binding pocket.

#### Analysis of RMSF

The root mean square fluctuation (RMSF) analysis was used to examine the residue-wise protein backbone flexibility and dynamic behavior during ligand binding throughout the 500 ns molecular dynamics simulation. RMSF measures the mean positional fluctuation of the individual residues around their mean positions over time with lower RMSF values, a structure is more rigid and higher value more flexible^92,97^. Figure 3C directly compares the RMSF profile of the protein complexing it with the chosen ligands with the standard compound (CID: 24958200). In general, the protein–ligand complexes showed low to moderate RMSF values of RMSF, which suggests that the backbone of the complexes does not undergo any major changes over the course of the simulation. The mean RMSF of L15 (CID: 9866696) (0.0958 nm), L08 (CID: 12313148) (0.0971 nm) were lower than the standard compound, 0.1214 nm, whereas L10 (CID: 318730) (0.1215 nm) was nearly similar to the standard compound and L03 (CID: 10425624) (0.1234 nm) was slightly higher. It is worth noting that L15 and L08 exhibited reduced mean values of RMSF as compared with the standard, indicating a better stabilization of the protein structure, whereas L10 was similar to the standard ligand in its RMSF behavior, and L03 showed slightly higher. These differences are further emphasized by the highest values of RMSF values. The standard compound had a maximum RMSF (0.5844 nm), while L10 showed the highest maximum RMSF value (0.5935 nm). L03 had a maximum RMSF value of 0.5502 nm, whereas L08 and L15 had lower maximum values of 0.4863 nm and 0.4616 nm, respectively, and the majority of these fluctuations were located in the terminal and loop regions. L10 on the other hand had the largest maximum RMSF value indicating higher flexibility at certain residues. The maximum RMSF of L03 (0.5502 nm) was lower than the standard compound, and it also indicates restrained fluctuations of residues. The lowest RMSF values of all complexes were uniformly low (~ 0.0389–0.0462 nm), which is within the range of rigid secondary structural elements, a- helices, and b- sheets. The residue level analysis indicated that the greatest variation was limited to loop regions and the N- and C-terminal residues and the residues in the structured core of the protein were fairly inflexible in all the complexes. Notably, ligands L15 and L08 caused the overall residue flexibility to decrease as compared to the standard, which means that the protein is better stabilized structurally after binding. Altogether, the analysis of RMSF indicates that L15 and L08 are more effective than the standard compound as they cause smaller averages of residue fluctuations, whereas L10 behaved similarly to the standard ligand and L03 showed slightly higher overall flexibility rather than being similar to the standard. The results also confirm that L15 and L08 are better choices and L10 is a similar alternative to the standard drug, in terms of structural stability of the proteins under dynamic conditions. Since PARP-1 inhibition depends on stable recognition within the catalytic pocket, reduced or controlled residue-level fluctuation. The RMSF result signifies that the best-performing ligands did not induce excessive flexibility in the catalytic domain, particularly in the structured core regions.

#### Analysis of radius of gyration

A major structural parameter that indicates the compactness and stability of the overall folding of a protein during molecular dynamics simulations is the radius of gyration (Rg). Smaller Rg values are a sign of a compact and structurally stable protein conformation, whereas larger ones are an indication of an expanded or flexible structure^98,99^. The Rg profiles for the protein complexes with the corresponding ligands (L03, L08, L10, and L15) were measured, and a direct comparison was made with the standard (CID: 24958200) during the 500 ns MD simulation, which is presented in Fig. 3D. The average Rg value in the standard complex was 2.070 nm, with a range of 2.014 to 2.171 nm, which has a moderate structural compactness with observable conformational breathing with time. Comparatively, L15 (CID: 9866696) had the lower mean Rg value of 2.050 nm, which implies that the protein is more compact and structurally stable compared with the standard. Equally, L10 (CID: 318730) exhibited a lower mean Rg of 2.061 nm, which means that the protein packing was somewhat tighter than that of the standard complex. The average Rg of the L03 (CID: 10425624) complex was 2.077 nm, slightly higher than that of the standard, indicating similar compactness and conformational stability. Similarly, L08 (CID: 12313148) showed an Rg mean of 2.048 nm as the Rg with stability at the end of the simulation, which was always smaller than the standard compound and, thus, showed stable folding behavior. Temporal analysis showed that all ligand-bound systems had comparatively steady Rg trajectories with few variations, but mainly related to loops and terminal residues. It is worth noting that the standard compound demonstrated a wider Rg range, but L15, L08 and L10 exhibited smaller Rg distributions, indicating that conformational variability upon ligand binding became smaller. Altogether, the Rg analysis shows that L15 and L08 are more effective at stabilizing the protein conformation in a compact form than the standard compound, whereas L10 also showed better Rg behavior than the standard, and L03 had similar behavior to the standard. These findings again argue in favor of L15 and L08 as better candidates and L03 and L10 as similar ones in preserving protein structural integrity under dynamic conditions. This suggests that the selected anthraquinones maintained the folded integrity of the PARP-1 catalytic domain and did not induce major conformational expansion during the simulation.

#### Analysis of SASA

Solvent accessible surface area (SASA) is the area of a protein surface that is accessible to solvent molecules and is an important measure of the compactness of the protein, its ability to fold, and conformational changes associated with the binding of ligands^92–94^. The changes in SASA during molecular dynamics simulations can be useful for understanding the structural rearrangement caused by ligand binding as well as the dynamical exposure of the residues to the solvent environment. The profiles of SASA were evaluated during the 500 ns MD window of the protein complex involving the chosen ligands and were simply compared with the standard compound (CID: 24958200) as represented in Fig. 3E. The average SASA value of the standard protein–ligand complex was about 173.88 nm^2^, which implies a greater extent of solvent-exposed protein surface and more flexibility of the protein–ligand complex during the simulation. Conversely, L15 (CID: 9866696) and L08 (CID: 12313148) demonstrated significantly lower average SASA values of approximately 171.31 nm^2^ and 170.76 nm^2^ on the average, respectively, indicating greater degree of protein compactness and reduced protein solvent exposure compared to the standard. These lower SASA values show that binding of L15 and L08 facilitates a more stable and tighter-packed protein conformation. Likewise, the average SASA of L03 (CID: 10425624) was around 173.15 nm^2^, which is slightly lower than the standard compound, but the observed value indicates good structural stability. L10 (CID: 318730) had a marginally greater average SASA of approximately 174.45 nm^2^, indicating controlled solvent exposure and preserved folding integrity. Temporal analysis indicated that standard complex exhibited greater SASA deviation, which indicated greater surface exposure and range of flexibility during the simulation. Conversely, ligand-bound complexes, in particular, L15, and L08, had smaller SASA distributions, suggesting smaller structural breathing and better conformational stability. On the whole, SASA analysis shows that L15 and L08 are better than the standard compound in terms of reduced solvent exposure and increasing protein compactness, and L03 is similar to the standard and L10 shows slightly higher SASA than the standard. These results also indicate that L15 and L08 are better candidates, and L03 is a similar alternative, which enhances their candidature as stable drug leads in dynamic physiological conditions^93^. Overall, the SASA analysis indicates that L15 and L08 produced lower solvent exposure and greater compactness of the PARP-1 catalytic domain than the standard compound, while L03 showed comparable behavior and L10 remained within an acceptable range. These findings suggest that selected anthraquinone derivatives, particularly L15 and L08, helped preserve a compact and stable protein conformation during the 500 ns simulation, supporting their relevance as dynamically stable PARP-1 binding candidates.

#### Analysis of energy profile

The total potential energy profile of the protein–ligand complexes during the **500 ns** molecular dynamics simulation is plotted as Fig. 3F, which is used to indicate equilibration and stability of the system’s energy^100^. All systems stabilized energy plateaus at early points in the simulation and had stable energy distributions throughout the production phase, which means they were well-equilibrated trajectories. The standard complex (CID: 24958200) had a relatively larger range in energy values and a wider band of varying values that indicate less favorable energetic stabilization. Conversely, lower and narrower-spread energy profiles were observed in ligand-bound complexes, indicating better stabilization upon ligand binding. LIG10 (CID: 318730) preserved the most negative overall energy levels throughout the simulation. However, L15 (CID: 9866696) and L03 (CID: 10425624) had the most stable energy curves with the least deviation, which suggests stronger and more stable protein–ligand interactions. The energetically stable behavior was also seen for L08 (CID: 12313148) and L10 (CID: 318730), which could preserve their lower energy levels than the standard during the simulation. Overall, the SASA analysis indicates that L15 and L08 produced lower solvent exposure and greater compactness of the PARP-1 catalytic domain than the standard compound, while L03 showed comparable behavior and L10 remained within an acceptable range. These findings suggest that selected anthraquinone derivatives, particularly L15 and L08, helped preserve a compact and stable protein conformation during the 500 ns simulation, supporting their relevance as dynamically stable PARP-1 binding candidates.

#### Analysis of hydrogen bonds

Hydrogen bonds play a vital role in protein folding, structure stability, and molecular recognition and the stability of hydrogen bonds throughout the molecular dynamics simulation is a good predictor of stable protein–ligand interactions^101,102^. The studies of hydrogen bond formation allow us to understand the strength of ligand binding, selectivity, and pharmacological behavior in general^92^. The use of hydrogen bond analysis in this study was conducted using a 500 ns MD simulation of the protein complexes bound with the chosen ligands and was compared directly with the standard compound (CID: 24958200). Figure 3G displays the time-dependency of hydrogen bonds. During the simulation, the number of hydrogen bonds in all the ligand-bound systems remained constant, which showed that these systems interacted consistently in the binding pocket. The standard compound (CID: 24958200) exhibited the weakest interaction profile, maintaining a very low average of fewer than 1 hydrogen bond and experiencing frequent periods of zero contact over the 500 ns timeframe. It is worth noting that L03 (CID: 10425624) and L15 (CID: 9866696) had significantly higher average numbers of hydrogen bonding, with L15 maintaining the highest interaction density (averaging ~ 3.75 bonds and peaking at 8), indicating stronger and more persistent intermolecular interactions than the standard ligand. Likewise, L08 (CID: 12313148) and L10 (CID: 318730) showed an average number of hydrogen bonds slightly higher than that of the standard compound, showing adequate binding stability. The number of hydrogen bonds in each of the complexes varied within a comparatively small range (between 0 and 10 bonds) but displayed dynamic and stable behavior in terms of bonding over the period of the simulation. Notably, the top ligand-bound systems did not show a sustained loss of hydrogen bonding, indicating that the ligands remained consistently engaged within the PARP-1 binding pocket without major binding-site destabilization. On the whole, the application of hydrogen bond analysis identified L15 and L03 as the best options, because they create more stable and dense hydrogen-bonds networks compared to the standard compound, and L08 shows the same behavior in terms of hydrogen bonding. These persistent interactions provide a mechanistic basis for the observed complex stability and support the prioritization of L03 and L15 as dynamically stable PARP-1 inhibitor leads for further experimental validation.

#### Analysis of molecular surface area (MolSA)

During the molecular dynamics simulation, molecular surface area (MolSA) analysis was conducted to evaluate changes in protein surface packing and compactness in response to ligand binding^103,104^. MolSA reflects changes in the exposed molecular surface of the protein and provides insight into ligand-induced conformational rearrangements, packing efficiency, and structural stability^9^. The MolSA patterns of the protein complexed with the ligands of interest were followed throughout the 500 ns simulation and a direct comparison with the standard compound (CID: 24958200) was made as shown in Fig. 3H. The standard complex exhibited a significant temporal variation in MolSA during the course of simulation indicating that the protein structure underwent continuous surface rearrangements and moderately breathing conformational changes. Such behavior is also characteristic of a dynamically flexible system and can be used to standard the evaluation of ligand-induced stabilization. Compared to them, L03 (CID: 10425624) and L15 (CID: 9866696) had more stable and less variable MolSA profiles and lower flattening amplitudes than those of the standard compound. This decrease in variability indicates greater surface packing density and also a higher level of structural compactness, meaning that these ligands are more effective at stabilizing the protein architecture throughout the simulation. The MolSA behavior of the L08 (CID: 12313148) complex was highly similar to that of the standard without causing marked expansion or reduction in surface exposure. This means that L08 maintains the intrinsic structural dynamics of the protein at an acceptable level of stability. Conversely, L10 (CID: 318730) demonstrated relatively higher MolSA fluctuations, which may indicate an increase in the exposure of the surface and a decrease in packing efficiency. This finding correlates with its increased RMSD, extended PCA distribution, and weaker DCCM correlation patterns, which means poorer dynamic stabilization compared to the standard compound. MolSA analysis showed that L03 and L15 promoted tighter surface packing and lower conformational variability than the standard compound, while L08 maintained a comparable stability profile. In contrast, the higher MolSA fluctuation of L10 suggests less efficient surface packing and weaker dynamic stabilization. These findings support the MD-based ranking of L03 as the most promising candidate and L15 as a strong alternative for further evaluation as PARP-1 inhibitor lead.

#### Analysis of principal component

**Fig. 4:**
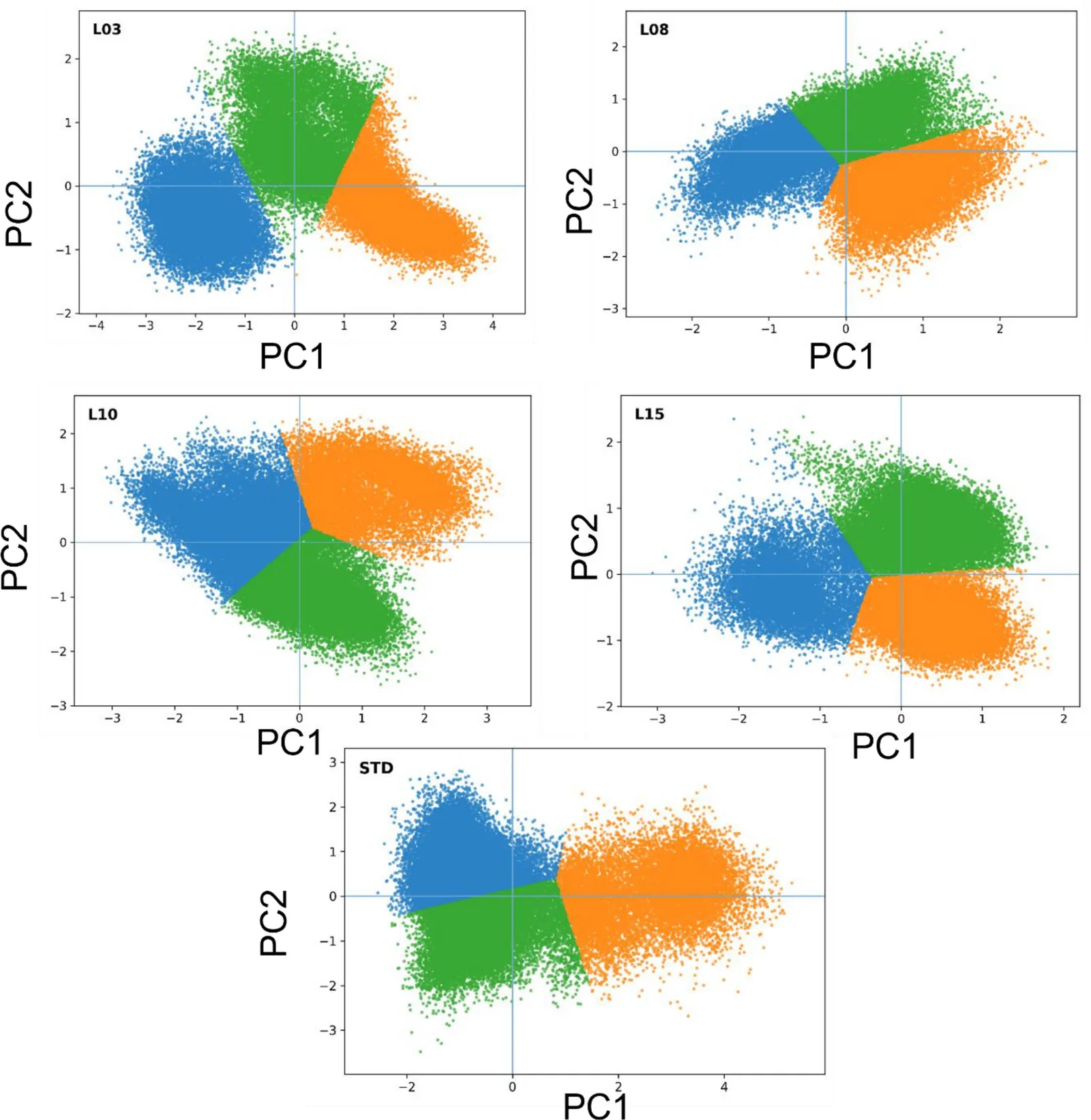
Principal component analysis (PCA) projections of PARP-1–ligand complexes during the 500 ns molecular dynamics simulation. The PC1–PC2 plots compare the conformational clustering and collective motions of PARP-1 complexes with L03, L08, L10, L15, and the standard compound (STD). PC1 and PC2 represent the first and second principal components of backbone motion, respectively; more compact clustering indicates restricted conformational sampling, whereas broader dispersion reflects greater protein motion during the simulation. PC1 = principal component 1 and PC2 = principal component 2.

Principal component analysis (PCA) of the backbone coordinates of the simulated complexes was conducted to reduce the number of dimensions of the molecular dynamics trajectories and to investigate the overall motions that govern protein–ligand dynamics^105–107^. PCA represents the major conformational motions of the system by mapping the trajectory onto the principal components (PCs), of which PC1 and PC2 capture the most important motions in the system on a global scale^90,108,109^. Figure 4 displays the two-dimensional PC1-PC2 projections of all the ligand–protein complexes including that of the standard compound (CID: 24958200) over the 500 ns trajectory.

The PCA map of the standard compound (CID: 24958200) shows a highly expanded distribution along PC1, which demonstrates the presence of multiple accessible conformational states of the protein and significant conformational flexibility at the time of the simulation. The conformational spread serves as the reference by which the dynamic behavior of the investigated ligands can be assessed. L03 (CID: 10425624) exhibited a distinct and well-clustered PCA distribution, particularly constrained along the PC2 axis, suggesting that the ligand restricted conformational sampling and improved the dynamic stability of the complex. The decrease in the spread along PC2 suggests that L03 successfully stabilizes the protein structure and constrains the large-scale collective movements. Similarly, L15 (CID: 9866696) had a distinct and highly restricted conformational cluster, and with transitions between restricted conformational substates. Such behavior indicates a balanced dynamic profile in which adequate flexibility is maintained without extreme structural fluctuations. L15 also shows superior conformational control in comparison to the standard compound. L10 (CID: 318730) on the contrary appeared to be more widely distributed among the major components indicating greater conformational variability and increased protein motion. This increased conformational space implies a low dynamic restraint compared to the top candidates, as also reflected in its less favorable RMSD, RMSF, and SASA profiles discussed above. An intermediate PCA pattern was observed for the L08 (CID: 12313148) complex, with moderate clustering and minimal dispersion in PC2. This behavior indicates that the complex is dynamically stable and neither too rigid nor too flexible. Collectively, PCA analysis over the 500 ns trajectory shows that L03 (CID: 10425624) and L15 (CID: 9866696) exhibited more restricted conformational sampling than the standard compound, indicating improved control of large-scale collective motions. In contrast, L10 (CID: 318730) showed greater conformational dispersion, suggesting weaker dynamic stabilization, whereas L08 (CID: 12313148) maintained an intermediate but acceptable stability profile. These findings support the MD-based prioritization of L03 as the strongest candidate and L15 as a promising alternative PARP-1 inhibitor lead.

#### Analysis of dynamic cross-correlation matrix (DCCM)

**Fig. 5:**
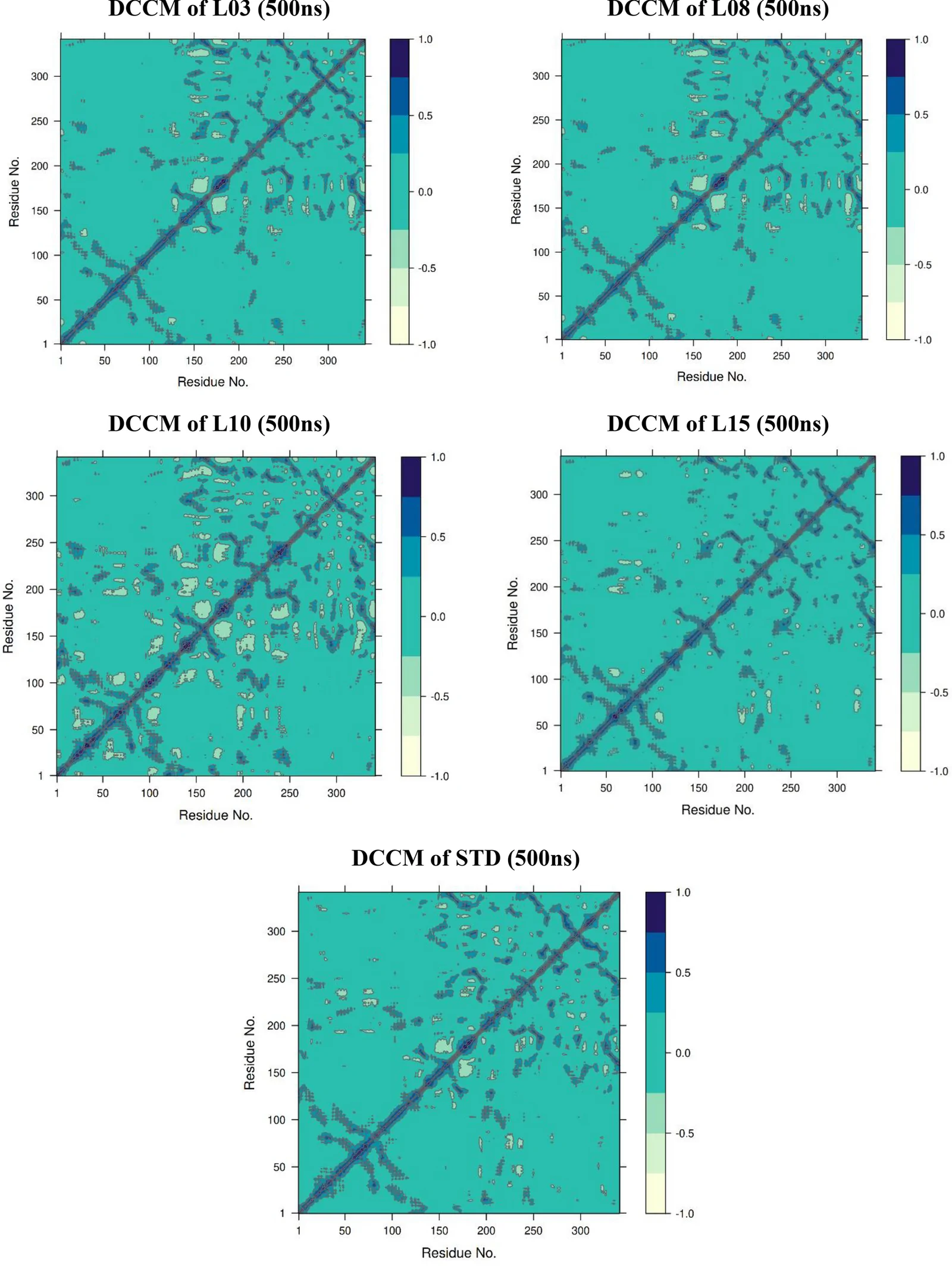
Dynamic cross-correlation matrix (DCCM) analysis of PARP-1 complexes with selected ligands and the standard compound over the 500 ns simulation. The maps show correlated and anti-correlated motions of Cα atoms across residue pairs for L03, L08, L10, L15, and STD. Positive values indicate coordinated residue motions, whereas negative values indicate opposite motions, providing insight into ligand-induced changes in PARP-1 dynamic behavior.

The analysis of the dynamic cross-correlation matrix (DCCM) of the correlated and anti-correlated motions of Cα atoms in the protein during the molecular dynamics simulation was conducted^110,111^. DCCM provides an understanding of long-range communication between residues in which positive associations (blue ones) indicate concerted motions whereas negative associations (yellow regions) reflect opposite motions. DCCM patterns can be compared to determine the effects of ligand binding on collective behavior in proteins^112^. Figure 5 shows the DCCM maps of all ligand–protein complexes in comparison with the standard ligand (CID: 24958200) over the extended 500 ns trajectory. The standard complex (CID: 24958200) displayed a predominantly positive pattern of correlated motions across the protein matrix, accompanied by strong correlated motions along the diagonal and sparse, weak off-diagonal anti-correlations, indicating intrinsic domain communication and highly restricted loop flexibility. This trend was used as a reference in assessing dynamic modulation caused by ligands. Among the studied compounds, the pattern of positive correlations in L03 (CID: 10425624) was stronger and more consistent, especially along off-diagonal regions, which suggests increased long-range communication between residues in this complex compared with the standard. At the same time, the size and distribution of anti-correlated motions were minimized, which indicates that L03 stabilizes cooperative motions in the protein and, consequently, facilitates a more dynamically synchronized system. Likewise, L15 (CID: 9866696) displayed clearly correlated motion networks with fewer disruptive anti-correlations as compared to the standard compound. The positive correlation areas are dominant, which indicates that L15 facilitates concerted structural motions, thereby contributing to better dynamic stability and regulated flexibility. Conversely, L10 (CID: 318730) had a lower intensity of correlation and a visibly greater extent of anti-correlated (light green) regions, which indicated less coordinated residue motions. This added dynamic heterogeneity implies that it is less efficient in maintaining intraprotein communication, as also reflected in its relatively poor performance in RMSD, RMSF, and PCA analyses compared to the standard. A DCCM pattern similar to that of the standard was observed for the L08 (CID: 12313148) complex, displaying widespread moderate correlated motions and very minimal anti-correlated regions of the structure. This implies that L08 does not induce excessive rigidity or destabilization of the protein but effectively maintains its inherent dynamic nature. Altogether, DCCM analysis showed that L03 and L15 produced stronger coordinated residue motions and fewer disruptive anti-correlations than the standard compound, indicating improved dynamic cooperativity within the PARP-1 catalytic domain. L08 displayed a correlation pattern comparable to the standard, whereas L10 showed weaker coordinated motion and greater dynamic heterogeneity. These findings are consistent with the PCA and other MD descriptors, further supporting L03 as the most promising candidate and L15 as a strong alternative for further evaluation as PARP-1 inhibitor leads.

## Conclusion

This study identified emodin-derived anthraquinones as promising computationally prioritized candidates for PARP-1 inhibition using an integrated computational approach, including biological activity prediction, drug-likeness assessment, ADMET profiling, molecular docking, and molecular dynamics simulations. The principal finding is that among the screened compounds, L03 (CID: 10425624) was the most promising lead, displaying a favorable balance of predicted biological activity, good ADME/T properties, strong binding affinity for the active site of PARP-1, and high dynamic stability throughout the simulation period. L03 showed stable interactions with crucial residues at the NAD+-binding site of PARP-1. Based on the MD simulations, the protein–ligand complex of L03 maintained a compact conformation with small fluctuations, supporting its potential as a dynamically stable PARP-1 binding candidate.

Further comparisons among the analogues studied showed that several of the compounds performed as well as or even better than the reference compound in some of the evaluated criteria. This observation supports the broader potential of the emodin scaffold for the development of PARP-1-targeted inhibitors. Specifically, L15 not only performed similarly to the reference compound in the studied criteria but also possessed favorable dynamic behavior and therefore can be considered as another potential lead. L08 showed dynamic behavior similar to that of the reference compound and a more favorable predicted oral/ADME profile. In contrast, although L10 had a strong docking score, its dynamic stability was significantly reduced. This observation emphasizes the importance of combining the docking score with post-docking pharmacokinetic parameters and molecular dynamics analysis for ranking the potential leads and indicates that docking affinity alone is insufficient for reliable lead prioritization.

Overall, the obtained results of this study support the potential of emodin-derived anthraquinones as PARP-1 inhibitors of interest for further studies, particularly with a view to their application in ovarian and prostate cancers, in where PARP-directed approach remains clinically relevant. Based on the integrated computational evidence, L03 appears to be the most promising alternative to the studied standard drug. Emodin and its derivatives appear to be promising scaffolds for the development of next-generation PARP-1 inhibitors. The main contribution of this work is the identification of L03 and related emodin-derived anthraquinones as structurally distinct lead candidates for further optimization. Experimental confirmation through in vitro and in vivo studies will be necessary to verify the predicted inhibitory activity, safety profile, and therapeutic value of the prioritized compounds.

### Limitations

This paper relies on computational analyses, such as molecular docking, ADME prediction and molecular dynamics simulations. Even though these methods are very informative, they may not fully capture biological complexity. Thus, the suggested compounds will require experimental validation through in vitro and in vivo testing, the preclinical and clinical evaluation of their efficacy, safety, and therapeutic applicability.

## Supplementary Information

Supplementary Information.

## Data Availability

Data are available under supplementary file section.

## Abbreviations

ADMET: Absorption, distribution, metabolism, excretion, and toxicity
BBB: Blood–brain barrier
CHB: Conventional hydrogen bond
CYP: Cytochrome P450
hERG: Human ether-à-go-go-related gene
MD: Molecular dynamics
MW: Molecular weight
PARP1: Poly (ADP-ribose) polymerase 1
PCA: Principal component analysis
RMSD: Root mean square deviation
RMSF: Root mean square fluctuation

## References

[1] Yao, Y. & Dai, W. Genomic instability and cancer. *J. Carcinog. Mutagen.* **5**, 1000165 (2014). 25541596 10.4172/2157-2518.1000165 PMC4274643

[2] Schiewer, M. J. & Knudsen, K. E. DNA damage response in prostate cancer. *Cold Spring Harb. Perspect. Med.* **9**, a030486 (2019). 29530944 10.1101/cshperspect.a030486 PMC6314076

[3] Liu, G. et al. Small molecule inhibitors targeting the cancers. *MedComm* **2022**(3), e181 (2020). 10.1002/mco2.181 PMC9560750 36254250

[4] Caron, M.-C. et al. Poly(ADP-ribose) polymerase-1 antagonizes DNA resection at double-strand breaks. *Nat. Commun.* **10**, 2954 (2019). 31273204 10.1038/s41467-019-10741-9 PMC6609622

[5] Fu, X. et al. Mechanism of PARP inhibitor resistance and potential overcoming strategies. *Genes Dis.* **11**, 306–320 (2023). 37588193 10.1016/j.gendis.2023.02.014 PMC10425807

[6] Rose, M. et al. PARP inhibitors: Clinical relevance, mechanisms of action and tumor resistance. *Front. Cell Dev. Biol.* **8**, 564601 (2020). 33015058 10.3389/fcell.2020.564601 PMC7509090

[7] Nizi, M. G., Sarnari, C. & Tabarrini, O. Privileged scaffolds for potent and specific inhibitors of mono-ADP-ribosylating PARPs. *Molecules* **28**, 5849 (2023). 37570820 10.3390/molecules28155849 PMC10420676

[8] Uddin, M. R. et al. Withaferin A and its derivatives as potential anti-cancer drug lead candidates: An in-silico drug design. *Dhaka Univ. J. Pharm. Sci.* **24**, 105–120 (2025).

[9] Uddin, M. R. et al. Identification of natural inhibitors targeting MMP-9 via network pharmacology and advanced molecular modeling approaches. *Next Res.* **5**, 101280 (2026).

[10] Sobuj, D. R. et al. Assessment of phytochemical screening, antimicrobial, antioxidant, and thrombolytic potential of ethanolic leaf extract of *Clerodendrum indicum*. *JPCI* **2**, 1–10 (2025).

[11] Chunarkar-Patil, P. et al. Anticancer drug discovery based on natural products: From computational approaches to clinical studies. *Biomedicines* **12**, 201 (2024). 38255306 10.3390/biomedicines12010201 PMC10813144

[12] Zhao, L. & Zheng, L. A review on bioactive anthraquinone and derivatives as the regulators for ROS. *Molecules* **28**, 8139 (2023). 38138627 10.3390/molecules28248139 PMC10745977

[13] Anjum, J. et al. Exploration of the GLUT-4 modulation potential of *Gynura procumbens* as a mechanism behind its antidiabetic activity. *Biomed. Pharmacol. J.* **17**, 2759–2768 (2024).

[14] Hsu, S.-C. & Chung, J.-G. Anticancer potential of emodin. *Biomedicine* **2**, 108–116 (2012). 32289000 10.1016/j.biomed.2012.03.003 PMC7104001

[15] Zheng, Q. et al. Advances in the study of emodin: An update on pharmacological properties and mechanistic basis. *Chin. Med.* **16**, 102 (2021). 34629100 10.1186/s13020-021-00509-z PMC8504117

[16] Yan, F. et al. In silico investigation of the molecular mechanism of PARP1 inhibition for the treatment of BRCA-deficient cancers. *Molecules* **28**, 1829 (2023). 36838818 10.3390/molecules28041829 PMC9961911

[17] Lee, Y.-R. et al. New approaches of PARP-1 inhibitors in human lung cancer cells and cancer stem-like cells by some selected anthraquinone-derived small molecules. *PLoS ONE* **8**, e56284 (2013). 23451039 10.1371/journal.pone.0056284 PMC3581553

[18] Yiu, C.-Y., Chiu, Y.-J. & Lin, T.-P. The ethyl acetate subfraction of *Polygonum cuspidatum* root containing emodin affect EBV gene expression and induce EBV-positive cells apoptosis. *Biol. Pharm. Bull.* **44**, 1837–1842 (2021). 34615812 10.1248/bpb.b21-00508

[19] Hwang, S. Y. et al. Emodin attenuates radioresistance induced by hypoxia in HepG2 cells via the enhancement of PARP1 cleavage and inhibition of JMJD2B. *Oncol. Rep.* **33**, 1691–1698 (2015). 25607726 10.3892/or.2015.3744

[20] Hollingsworth, S. A. & Dror, R. O. Molecular dynamics simulation for all. *Neuron* **99**, 1129–1143 (2018). 30236283 10.1016/j.neuron.2018.08.011 PMC6209097

[21] Aghajani, J. et al. Molecular dynamic simulations and molecular docking as a potential way for designed new inhibitor drug without resistance. *Tanaffos* **21**, 1–14 (2022). 36258912 PMC9571241

[22] Serrano, D. R. et al. Artificial intelligence (AI) applications in drug discovery and drug delivery: Revolutionizing personalized medicine. *Pharmaceutics* **16**, 1328 (2024). 39458657 10.3390/pharmaceutics16101328 PMC11510778

[23] Moldovan, O.-L. et al. Identification of some glutamic acid derivatives with biological potential by computational methods. *Molecules* **28**, 4123 (2023). 37241864 10.3390/molecules28104123 PMC10224408

[24] Lipinski, C. A. Lead- and drug-like compounds: The rule-of-five revolution. *Drug. Discov. Today. Technol.* **1**, 337–341 (2004). 24981612 10.1016/j.ddtec.2004.11.007

[25] Mikov, M. et al. The role of drug metabolites in the inhibition of Cytochrome P450 enzymes. *Eur. J. Drug Metab. Pharmacokinet.* **42**, 881–890 (2017). 28534261 10.1007/s13318-017-0417-y

[26] Feng, B. et al. Renal clearance in drug discovery and development: Molecular descriptors, drug transporters and disease state. *Expert Opin. Drug Metab. Toxicol.* **6**, 939–952 (2010). 20433402 10.1517/17425255.2010.482930

[27] Fuse, E. et al. Prediction of the maximal tolerated dose (MTD) and therapeutic effect of anticancer drugs in humans: Integration of pharmacokinetics with pharmacodynamics and toxicodynamics. *Cancer Treat. Rev.* **21**, 133–157 (1995). 7758004 10.1016/0305-7372(95)90024-1

[28] Basant, N., Gupta, S. & Singh, K. P. Predicting human intestinal absorption of diverse chemicals using ensemble learning based QSAR modeling approaches. *Comput. Biol. Chem.* **61**, 178–196 (2016). 26881740 10.1016/j.compbiolchem.2016.01.005

[29] Wei, M. et al. HobPre: Accurate prediction of human oral bioavailability for small molecules. *J. Cheminform.* **14**, 1 (2022). 34991690 10.1186/s13321-021-00580-6 PMC8740492

[30] Kim, M. T. et al. Critical evaluation of human oral bioavailability for pharmaceutical drugs by using various cheminformatics approaches. *Pharm. Res.* **31**, 1002–1014 (2014). 24306326 10.1007/s11095-013-1222-1 PMC3955412

[31] Panse, N. & Gerk, P. M. The Caco-2 Model: Modifications and enhancements to improve efficiency and predictive performance. *Int. J. Pharm.* **624**, 122004 (2022). 35820514 10.1016/j.ijpharm.2022.122004

[32] Wei, Y. et al. Investigation of *in silico* studies for cytochrome P450 isoforms specificity. *Comput. Struct. Biotechnol. J.* **23**, 3090–3103 (2024). 39188968 10.1016/j.csbj.2024.08.002 PMC11347072

[33] Fan, J. et al. Predicting elimination of small-molecule drug half-life in pharmacokinetics using ensemble and consensus machine learning methods. *J. Chem. Inf. Model.* **64**, 3080–3092 (2024). 38563433 10.1021/acs.jcim.3c02030

[34] Pettersen, E. F. et al. UCSF Chimera–a visualization system for exploratory research and analysis. *J. Comput. Chem.* **25**, 1605–1612 (2004). 15264254 10.1002/jcc.20084

[35] Butt, S. S. et al. Molecular docking using Chimera and Autodock Vina software for nonbioinformaticians. *JMIR Bioinform. Biotechnol.* **1**, e14232 (2020). 38943236 10.2196/14232 PMC11168238

[36] Zardecki, C. et al. RCSB Protein Data Bank: A resource for chemical, biochemical, and structural explorations of large and small biomolecules. *J. Chem. Educ.* **93**, 569–575 (2016).

[37] Huang, Y.-H., Rose, P. W. & Hsu, C.-N. Citing a data repository: A case study of the Protein Data Bank. *PLoS ONE* **10**, e0136631 (2015). 26317409 10.1371/journal.pone.0136631 PMC4552849

[38] Guterres, H. & Im, W. Improving protein-ligand docking results with high-throughput molecular dynamics simulations. *J. Chem. Inf. Model.* **60**, 2189–2198 (2020). 32227880 10.1021/acs.jcim.0c00057 PMC7534544

[39] Langelier, M.-F. et al. Structural basis for DNA-dependent poly(ADP-ribosyl)ation by human PARP-1. *Science* **336**, 728–732 (2012). 22582261 10.1126/science.1216338 PMC3532513

[40] Yang, Z. et al. UCSF Chimera, MODELLER, and IMP: An integrated modeling system. *J. Struct. Biol.* **179**, 269–278 (2012). 21963794 10.1016/j.jsb.2011.09.006 PMC3410985

[41] Wang, K. et al. How ligands regulate the binding of PARP1 with DNA: Deciphering the mechanism at the molecular level. *PLoS ONE* **18**, e0290176 (2023). 37582112 10.1371/journal.pone.0290176 PMC10426920

[42] Poroikov, V. V. et al. Computer-aided prediction of biological activity spectra for organic compounds: The possibilities and limitations. *Russ. Chem. Bull.* **68**, 2143–2154 (2019).

[43] Lagunin, A. et al. PASS: Prediction of activity spectra for biologically active substances. *Bioinformatics* **16**, 747–748 (2000). 11099264 10.1093/bioinformatics/16.8.747

[44] Stepanchikova, A. V. et al. Prediction of biological activity spectra for substances: Evaluation on the diverse sets of drug-like structures. *Curr. Med. Chem.* **10**, 225–233 (2003). 12570709 10.2174/0929867033368510

[45] Elayyan, A. E. M. et al. Identification of bioactive phytoconstituents as promising ABL2 inhibitors using virtual screening and molecular dynamics simulation. *Sci Rep* **15**, 44528 (2025). 41444785 10.1038/s41598-025-28195-z PMC12738542

[46] Suha, H. N. et al. Evaluating the anticancer properties of novel piscidinol a derivatives: Insights from DFT, molecular docking, and molecular dynamics studies. *ACS Omega* **9**, 49639–49661 (2024). 39713673 10.1021/acsomega.4c07808 PMC11656217

[47] Use of molecular docking computational tools in drug discovery. In *Progress in Medicinal Chemistry*, 273–343 (Elsevier). 10.1016/bs.pmch.2021.01.004 34147204

[48] Ferreira, L. et al. Molecular docking and structure-based drug design strategies. *Molecules* **20**, 13384–13421 (2015). 26205061 10.3390/molecules200713384 PMC6332083

[49] Trott, O. & Olson, A. J. AutoDock Vina: Improving the speed and accuracy of docking with a new scoring function, efficient optimization, and multithreading. *J. Comput. Chem.* **31**, 455–461 (2010). 19499576 10.1002/jcc.21334 PMC3041641

[50] Ryan, K. et al. Dissecting the molecular determinants of clinical PARP1 inhibitor selectivity for tankyrase1. *J. Biol. Chem.* 10.1074/jbc.RA120.016573 (2021). 33361107 PMC7948648

[51] Meng, E. C. et al. UCSF ChimeraX: Tools for structure building and analysis. *Protein Sci.* **32**, e4792 (2023). 37774136 10.1002/pro.4792 PMC10588335

[52] Khalil, F. & Läer, S. Physiologically based pharmacokinetic modeling: Methodology, applications, and limitations with a focus on its role in pediatric drug development. *J. Biomed. Biotechnol.* **2011**, 907461 (2011). 21716673 10.1155/2011/907461 PMC3118302

[53] Arango, J. P. B. et al. *In silico* evaluation of pharmacokinetic properties and molecular docking for the identification of potential anticancer compounds. *Comput. Biol. Chem.* **120**, 108626 (2026). 40803051 10.1016/j.compbiolchem.2025.108626

[54] Kim, S. et al. PubChem substance and compound databases. *Nucleic Acids Res.* **44**, D1202–D1213 (2016). 26400175 10.1093/nar/gkv951 PMC4702940

[55] Wu, F. et al. Computational approaches in preclinical studies on drug discovery and development. *Front. Chem.* **8**, 726 (2020). 33062633 10.3389/fchem.2020.00726 PMC7517894

[56] Feinstein, W. P. & Brylinski, M. Calculating an optimal box size for ligand docking and virtual screening against experimental and predicted binding pockets. *J. Cheminform.* **7**, 18 (2015). 26082804 10.1186/s13321-015-0067-5 PMC4468813

[57] Lee, S., Wang, D., Seeliger, M. A. et al. Calculating protein-ligand residence times through state predictive information bottleneck based enhanced sampling. *bioRxiv*. 2024.04.16.589710 (2024). 10.1021/acs.jctc.4c00503 PMC11990086 38991145

[58] Sawaran Singh, N. S. et al. Effect of atomic porosity on the mechanical properties of aluminium polycrystalline using molecular dynamics simulation. *Results Eng.* **25**, 104491 (2025).

[59] Cediel-Becerra, J. D. D., Silva, J. C. F. & Dias, R. EquilibraTor streamlines molecular dynamics simulations in a single execution. *Comput. Struct. Biotechnol. J.* **27**, 5318–5325 (2025). 41377122 10.1016/j.csbj.2025.11.034 PMC12686642

[60] Vidal-Limon, A., Aguilar-Toalá, J. E. & Liceaga, A. M. Integration of molecular docking analysis and molecular dynamics simulations for studying food proteins and bioactive peptides. *J. Agric. Food Chem.* **70**, 934–943 (2022). 34990125 10.1021/acs.jafc.1c06110

[61] Simmonett, A. C. & Brooks, B. R. A compression strategy for particle mesh Ewald theory. *J. Chem. Phys.* **154**, 054112 (2021). 33557541 10.1063/5.0040966 PMC7986272

[62] Hess, B. et al. LINCS: A linear constraint solver for molecular simulations. *J. Comput. Chem.* **18**, 1463–1472 (1997). 10.1021/ct700200b 26619985

[63] Jensen, L. E., Rao, S., Schuschnig, M. et al. Membrane curvature sensing and stabilization by the autophagic LC3 lipidation machinery. *Sci. Adv*. **8**, eadd1436. 10.1126/sciadv.add1436 PMC9750143 36516251

[64] Soliman, M. E. et al. Simulation models for prediction of bioavailability of medicinal drugs-the interface between experiment and computation. *AAPS PharmSciTech* **23**, 86 (2022). 35292867 10.1208/s12249-022-02229-5

[65] Brown, R. W. Abiotic scaffolds in medicinal chemistry: Not a waste of chemical space. *Future Med. Chem.* **13**, 211–224 (2021). 33445971 10.4155/fmc-2020-0294

[66] Pires, D. E. V., Blundell, T. L. & Ascher, D. B. pkCSM: Predicting small-molecule pharmacokinetic and toxicity properties using graph-based signatures. *J. Med. Chem.* **58**, 4066–4072 (2015). 25860834 10.1021/acs.jmedchem.5b00104 PMC4434528

[67] Myung, Y., de Sá, A. G. C. & Ascher, D. B. Deep-PK: Deep learning for small molecule pharmacokinetic and toxicity prediction. *Nucleic Acids Res.* **52**, W469–W475 (2024). 38634808 10.1093/nar/gkae254 PMC11223837

[68] Sultana, A. et al. Exploring the therapeutic potential of *Premna obtusifolia* and *Oxalis corniculata*: A combined in vitro bioassay and molecular docking study. *JMNP* **3**, 100003 (2026).

[69] Sevin, E. et al. Accelerated Caco-2 cell permeability model for drug discovery. *J. Pharmacol. Toxicol. Methods* **68**, 334–339 (2013). 23916595 10.1016/j.vascn.2013.07.004

[70] Kus, M., Ibragimow, I. & Piotrowska-Kempisty, H. Caco-2 cell line standardization with pharmaceutical requirements and in vitro model suitability for permeability assays. *Pharmaceutics* **15**, 2523 (2023). 38004503 10.3390/pharmaceutics15112523 PMC10674574

[71] Niazi, S. K. Non-invasive drug delivery across the blood–brain barrier: A prospective analysis. *Pharmaceutics* **15**, 2599 (2023). 38004577 10.3390/pharmaceutics15112599 PMC10674293

[72] Watanabe, R. et al. Predicting fraction unbound in human plasma from chemical structure: Improved accuracy in the low value ranges. *Mol. Pharm.* **15**, 5302–5311 (2018). 30259749 10.1021/acs.molpharmaceut.8b00785

[73] Davies, M. et al. Improving the accuracy of predicted human pharmacokinetics: Lessons learned from the AstraZeneca drug pipeline over two decades. *Trends Pharmacol. Sci.* **41**, 390–408 (2020). 32359836 10.1016/j.tips.2020.03.004

[74] Decoding the Role of CYP450 Enzymes in Metabolism and Disease: A Comprehensive Review, https://www.mdpi.com/2227-9059/12/7/1467 (Accessed 10 Jan 2026). 10.3390/biomedicines12071467 PMC11275228 39062040

[75] Deodhar, M. et al. Mechanisms of CYP450 inhibition: Understanding drug-drug interactions due to mechanism-based inhibition in clinical practice. *Pharmaceutics* **12**, 846 (2020). 32899642 10.3390/pharmaceutics12090846 PMC7557591

[76] Kido, Y., Matsson, P. & Giacomini, K. M. Profiling of a prescription drug library for potential renal drug-drug interactions mediated by the organic cation transporter 2. *J. Med. Chem.* **54**, 4548–4558 (2011). 21599003 10.1021/jm2001629 PMC3257218

[77] Harper, J. N. & Wright, S. H. Multiple mechanisms of ligand interaction with the human organic cation transporter, OCT2. *Am. J. Physiol. Renal Physiol.* **304**, F56–F67 (2013). 23034939 10.1152/ajprenal.00486.2012 PMC3971011

[78] Smith, D. A. et al. Relevance of half-life in drug design. *J. Med. Chem.* **61**, 4273–4282 (2018). 29112446 10.1021/acs.jmedchem.7b00969

[79] Silva, C. H. T. P. & Taft, C. A. ADMET properties, database screening, molecular dynamics, density functional, and docking studies of novel potential anti-cancer compounds. *J. Biomol. Struct. Dyn.* **24**, 263–268 (2006). 17054384 10.1080/07391102.2006.10507118

[80] Xu, M. et al. In silico prediction of chemical aquatic toxicity by multiple machine learning and deep learning approaches. *J. Appl. Toxicol.* **42**, 1766–1776 (2022). 35653511 10.1002/jat.4354

[81] Sun, L. et al. In silico prediction of chemical aquatic toxicity with chemical category approaches and substructural alerts. *Toxicol. Res.* **4**, 452–463 (2015).

[82] David, S. & Hamilton, J. P. Drug-induced liver injury. *US Gastroenterol. Hepatol. Rev.* **6**, 73–80 (2010). 21874146 PMC3160634

[83] Saleh, A. K. et al. Exploring drug-induced liver injury: Comprehensive insights into mechanisms and management of hepatotoxic agents. *Futur. J. Pharm. Sci.* **11**, 38 (2025).

[84] Gajewicz-Skretna, A. et al. Generating accurate *in silico* predictions of acute aquatic toxicity for a range of organic chemicals: Towards similarity-based machine learning methods. *Chemosphere* **280**, 130681 (2021). 34162070 10.1016/j.chemosphere.2021.130681

[85] Antolin, A. A. et al. Exploring the effect of PARP-1 flexibility in docking studies. *J. Mol. Graph. Model.* **45**, 192–201 (2013). 24056306 10.1016/j.jmgm.2013.08.006

[86] Wang, H. Prediction of protein–ligand binding affinity via deep learning models. *Brief. Bioinform.* **25**, bbae081 (2024). 38446737 10.1093/bib/bbae081 PMC10939342

[87] Do, P.-C., Lee, E. H. & Le, L. Steered molecular dynamics simulation in rational drug design. *J. Chem. Inf. Model.* **58**, 1473–1482 (2018). 29975531 10.1021/acs.jcim.8b00261

[88] Salo-Ahen, O. M. H. et al. Molecular dynamics simulations in drug discovery and pharmaceutical development. *Processes* **9**, 71 (2020).

[89] Uhomoibhi, J. O. et al. Molecular modeling identification of potential drug candidates from selected African plants against SARS-CoV-2 key druggable proteins. *Sci. Afr.* **17**, e01279 (2022). 35856008 10.1016/j.sciaf.2022.e01279 PMC9279166

[90] David, C. C. & Jacobs, D. J. Principal component analysis: A method for determining the essential dynamics of proteins. *Methods Mol. Biol.* **1084**, 193–226 (2014). 24061923 10.1007/978-1-62703-658-0_11 PMC4676806

[91] Velázquez-Libera, J. L. et al. LigRMSD: A web server for automatic structure matching and RMSD calculations among identical and similar compounds in protein-ligand docking. *Bioinformatics* **36**, 2912–2914 (2020). 31926012 10.1093/bioinformatics/btaa018

[92] da Fonseca, A. M. et al. Screening of potential inhibitors targeting the main protease structure of SARS-CoV-2 via molecular docking, and approach with molecular dynamics, RMSD, RMSF, H-Bond, SASA and MMGBSA. *Mol. Biotechnol.* **66**, 1919–1933 (2024). 37490200 10.1007/s12033-023-00831-x

[93] Konstantinidis, K. et al. On the estimation of the molecular inaccessible volume and the molecular accessible surface of a ligand in protein–ligand systems. *Mol. Syst. Des. Eng.* **6**, 946–963 (2021).

[94] Marsh, J. A. & Teichmann, S. A. Relative solvent accessible surface area predicts protein conformational changes upon binding. *Structure* **19**, 859–867 (2011). 21645856 10.1016/j.str.2011.03.010 PMC3145976

[95] Shukla, R. & Tripathi, T. Molecular dynamics simulation of protein and protein–ligand complexes. In *Computer-Aided Drug Design* (ed Singh, D. B. ed) 133–161 (Springer).

[96] Sargsyan, K., Grauffel, C. & Lim, C. How molecular size impacts RMSD applications in molecular dynamics simulations. *J. Chem. Theory Comput.* **13**, 1518–1524 (2017). 28267328 10.1021/acs.jctc.7b00028

[97] Adelusi, T. I. et al. Molecular modeling in drug discovery. *Inform. Med. Unlocked* **29**, 100880 (2022).

[98] Tanner, J. J. Empirical power laws for the radii of gyration of protein oligomers. *Acta Crystallogr. D. Struct. Biol.* **72**, 1119–1129 (2016). 27710933 10.1107/S2059798316013218 PMC5053138

[99] Lobanov, MYu., Bogatyreva, N. S. & Galzitskaya, O. V. Radius of gyration as an indicator of protein structure compactness. *Mol. Biol.* **42**, 623–628 (2008). 18856071

[100] Deng, Y. & Roux, B. Computations of standard binding free energies with molecular dynamics simulations. *J. Phys. Chem. B.* **113**, 2234–2246 (2009). 19146384 10.1021/jp807701h PMC3837708

[101] Sethi, A., Agrawal, N. & Brezovsky, J. Impact of water models on the structure and dynamics of enzyme tunnels. *Comput. Struct. Biotechnol. J.* **23**, 3946–3954 (2024). 39582894 10.1016/j.csbj.2024.10.051 PMC11584523

[102] Bitencourt-Ferreira, G., Veit-Acosta, M. & de Azevedo, W. F. Hydrogen bonds in protein-ligand complexes. *Methods. Mol. Biol.* **2053**, 93–107 (2019). 31452101 10.1007/978-1-4939-9752-7_7

[103] Faruque, M. et al. Investigating small molecules in propolis as Nipah virus glycoprotein (NiV-G) inhibitors through molecular interaction studies. *Heliyon* **11**, e42595 (2025). 40051842 10.1016/j.heliyon.2025.e42595 PMC11883394

[104] Pereira, G. R. C. et al. In silico analysis of the tryptophan hydroxylase 2 (TPH2) protein variants related to psychiatric disorders. *PLoS ONE* **15**, e0229730 (2020). 32119710 10.1371/journal.pone.0229730 PMC7051086

[105] Moradi, S. et al. A review on description dynamics and conformational changes of proteins using combination of principal component analysis and molecular dynamics simulation. *Comput. Biol. Med.* **183**, 109245 (2024). 39388840 10.1016/j.compbiomed.2024.109245

[106] Giuliani, A. The application of principal component analysis to drug discovery and biomedical data. *Drug Discov. Today* **22**, 1069–1076 (2017). 28111329 10.1016/j.drudis.2017.01.005

[107] Maisuradze, G. G., Liwo, A. & Scheraga, H. A. Principal component analysis for protein folding dynamics. *J. Mol. Biol.* **385**, 312–329 (2009). 18952103 10.1016/j.jmb.2008.10.018 PMC2652707

[108] Salamat, A. et al. SAR, molecular docking and molecular dynamic simulation of natural inhibitors against SARS-CoV-2 Mpro spike protein. *Molecules* **29**, 1144 (2024). 38474656 10.3390/molecules29051144 PMC10934104

[109] Hansen, M. et al. pcaGoPromoter–An R package for biological and regulatory interpretation of principal components in genome-wide gene expression data. *PLoS ONE* **7**, e32394 (2012). 22384239 10.1371/journal.pone.0032394 PMC3288097

[110] dos Santos Nascimento, I. J. et al. Dynamic cross-correlation matrix (DCCM) reveals new insights to discover new NLRP3 inhibitors useful as anti-inflammatory drugs. *Med. Sci. Forum* **14**, 84 (2022).

[111] Kumari, M., Singh, R. & Subbarao, N. Exploring the interaction mechanism between potential inhibitor and multi-target Mur enzymes of *Mycobacterium tuberculosis* using molecular docking, molecular dynamics simulation, principal component analysis, free energy landscape, dynamic cross-correlation matrices, vector movements, and binding free energy calculation. *J. Biomol. Struct. Dyn.* **40**, 13497–13526 (2022). 34662260 10.1080/07391102.2021.1989040

[112] Yu, H. & Dalby, P. A. A beginner’s guide to molecular dynamics simulations and the identification of cross-correlation networks for enzyme engineering. In *Methods in Enzymology*, 15–49 (Elsevier). 10.1016/bs.mie.2020.04.020 32896280

[113] Teo, M. T. W. et al. The role of microRNA-binding site polymorphisms in DNA repair genes as risk factors for bladder cancer and breast cancer and their impact on radiotherapy outcomes. *Carcinogenesis* **33**, 581–586 (2012). 22166496 10.1093/carcin/bgr300 PMC3291859

[114] Wang, M. et al. Genetic variants of XRCC1, APE1, and ADPRT genes and risk of bladder cancer. *DNA Cell Biol.* **29**, 303–311 (2010). 20218899 10.1089/dna.2009.0969

[115] Liang, B. et al. Synergistic suppressive effect of PARP-1 inhibitor PJ34 and HDAC inhibitor SAHA on proliferation of liver cancer cells. *J. Huazhong Univ. Sci. Technol. [Med Sci]* **35**, 535–540 (2015). 10.1007/s11596-015-1466-6 26223923

[116] Xu, M. et al. Landscape analysis of lncRNAs shows that *DDX11-AS1* promotes cell-cycle progression in liver cancer through the PARP1/*p53* axis. *Cancer Lett.* **520**, 282–294 (2021). 34371129 10.1016/j.canlet.2021.08.001

[117] Cherng, Y.-G. et al. Induced mitochondrial alteration and DNA damage via IFNGR-JAK2-STAT1-PARP1 pathway facilitates viral hepatitis associated hepatocellular carcinoma aggressiveness and stemness. *Cancers* **13**, 2755 (2021). 34199353 10.3390/cancers13112755 PMC8199505

[118] Gong, R. et al. Identification and evaluation of a novel PARP1 inhibitor for the treatment of triple-negative breast cancer. *Chem. Biol. Interact.* **382**, 110567 (2023). 37271214 10.1016/j.cbi.2023.110567

[119] Yang, T. et al. The PRMT6/PARP1/CRL4B complex regulates the circadian clock and promotes breast tumorigenesis. *Adv. Sci.* **10**, 2202737 (2023). 10.1002/advs.202202737 PMC10190619 36941223

[120] Li, X. et al. Dual target PARP1/EZH2 inhibitors inducing excessive autophagy and producing synthetic lethality for triple-negative breast cancer therapy. *Eur. J. Med. Chem.* **265**, 116054 (2024). 38134746 10.1016/j.ejmech.2023.116054

[121] Long, L.-L. et al. PARP inhibition induces synthetic lethality and adaptive immunity in LKB1-mutant lung cancer. *Cancer Res.* **83**, 568–581 (2023). 36512628 10.1158/0008-5472.CAN-22-1740

[122] Juncheng, P. et al. PARP1 inhibition elicits immune responses against non-small cell lung cancer. *Oncoimmunology* **11**, 2111915 (2022). 35979387 10.1080/2162402X.2022.2111915 PMC9377245

[123] Roggero, C. M. et al. CDK4/6 inhibitors promote PARP1 degradation and synergize with PARP inhibitors in non-small cell lung cancer. *Transl. Oncol.* **52**, 102231 (2025). 39662449 10.1016/j.tranon.2024.102231 PMC11683282

[124] Ji, X. et al. PD-L1, PARP1, and MMRs as potential therapeutic biomarkers for neuroendocrine cervical cancer. *Cancer Med.* **10**, 4743–4751 (2021). 34076351 10.1002/cam4.4034 PMC8290238

[125] Rajawat, J. & Banerjee, M. Poly(ADP-ribose) polymerase1 (PARP1) and PARP inhibitors: New frontiers in cervical cancer. *Biochem. Biophys. Res. Commun.* **738**, 150943 (2024). 39504715 10.1016/j.bbrc.2024.150943

[126] IJff, M. et al. PARP1-inhibition sensitizes cervical cancer cell lines for chemoradiation and thermoradiation. *Cancers* **13**, 2092 (2021). 33926008 10.3390/cancers13092092 PMC8123631

[127] Wang, F. et al. PARP1 upregulation in recurrent oral cancer and treatment resistance. *Front. Cell Dev. Biol.* 10.3389/fcell.2021.804962 (2022). 35071239 PMC8769238

[128] Kossatz, S., Weber, W. & Reiner, T. Detection and delineation of oral cancer with a PARP1-targeted optical imaging agent. *Mol. Imaging* **16**, 1536012117723786 (2017). 28856922 10.1177/1536012117723786 PMC5582799

[129] Gunderson, C. C. & Moore, K. N. Olaparib: An oral PARP-1 and PARP-2 inhibitor with promising activity in ovarian cancer. *Future Oncol.* **11**, 747–757 (2015). 25757679 10.2217/fon.14.313

[130] Vaculová, A. et al. Tumor necrosis factor-alpha induces apoptosis associated with poly(ADP-ribose) polymerase cleavage in HT-29 colon cancer cells. *Anticancer Res.* **22**, 1635–1639 (2002). 12168847

[131] Schwab, M. et al. PPARgamma is involved in mesalazine-mediated induction of apoptosis and inhibition of cell growth in colon cancer cells. *Carcinogenesis* **29**, 1407–1414 (2008). 18544567 10.1093/carcin/bgn118

[132] Zheng, B., Chai, R. & Yu, X. Downregulation of NIT2 inhibits colon cancer cell proliferation and induces cell cycle arrest through the caspase-3 and PARP pathways. *Int. J. Mol. Med.* **35**, 1317–1322 (2015). 25738796 10.3892/ijmm.2015.2125

[133] Wu, J. et al. PARP1-stabilised FOXQ1 promotes ovarian cancer progression by activating the LAMB3/WNT/β-catenin signalling pathway. *Oncogene* **43**, 866–883 (2024). 38297082 10.1038/s41388-024-02943-3

[134] Xiao, M. et al. Let-7e suppresses DNA damage repair and sensitizes ovarian cancer to Cisplatin through targeting PARP1. *Mol. Cancer Res.* **18**, 436–447 (2020). 31722968 10.1158/1541-7786.MCR-18-1369

[135] Zuo, W.-W. et al. High expression of PARP1 in tumor and stroma cells predicts different prognosis and platinum resistance in patients with advanced epithelial ovarian cancer. *Front. Oncol.* 10.3389/fonc.2022.931445 (2022). 35875162 PMC9301997

[136] Zhang, Y. et al. Macrocarpal I induces immunogenic cell death and synergizes with immune checkpoint inhibition by targeting tubulin and PARP1 in colorectal cancer. *Cell Death Discov.* **11**, 73 (2025). 39987121 10.1038/s41420-025-02360-9 PMC11846858

[137] Jing, Z. et al. NCAPD2 inhibits autophagy by regulating Ca2+/CAMKK2/AMPK/mTORC1 pathway and PARP-1/SIRT1 axis to promote colorectal cancer. *Cancer Lett.* **520**, 26–37 (2021). 34229059 10.1016/j.canlet.2021.06.029

[138] Wang, J. et al. FTO promotes colorectal cancer progression and chemotherapy resistance via demethylating G6PD/PARP1. *Clin. Transl. Med.* **12**, e772 (2022). 35297218 10.1002/ctm2.772 PMC8926902

[139] Quiñonero, F. et al. PARP1 inhibition by Olaparib reduces the lethality of pancreatic cancer cells and increases their sensitivity to Gemcitabine. *Biomed. Pharmacother.* **155**, 113669 (2022). 36113257 10.1016/j.biopha.2022.113669

[140] Zhu, H. et al. PARP inhibitors in pancreatic cancer: Molecular mechanisms and clinical applications. *Mol. Cancer* **19**, 49 (2020). 32122376 10.1186/s12943-020-01167-9 PMC7053129

[141] Viera, T. & Patidar, P. L. DNA damage induced by KP372-1 hyperactivates PARP1 and enhances lethality of pancreatic cancer cells with PARP inhibition. *Sci. Rep.* **10**, 20210 (2020). 33214574 10.1038/s41598-020-76850-4 PMC7677541

[142] Zhou, R. et al. PARP1 rs1136410 C/C genotype associated with an increased risk of esophageal cancer in smokers. *Mol. Biol. Rep.* **48**, 1485–1491 (2021). 33528729 10.1007/s11033-021-06169-4

[143] Zhou, R.-M. et al. PARP1 gene polymorphisms and the prognosis of esophageal cancer patients from Cixian high-incidence region in Northern China. *Asian Pac. J. Cancer Prev.* **21**, 2987–2992 (2020). 33112558 10.31557/APJCP.2020.21.10.2987 PMC7798169

[144] Nasuno, T. et al. Effect of a poly(ADP-ribose) polymerase-1 inhibitor against esophageal squamous cell carcinoma cell lines. *Cancer Sci.* **105**, 202–210 (2014). 24219164 10.1111/cas.12322 PMC4317825

[145] Dehdashti, F. et al. Pilot study: PARP1 imaging in advanced prostate cancer. *Mol. Imaging Biol.* **24**, 853–861 (2022). 35701722 10.1007/s11307-022-01746-w PMC9681698

[146] Lai, Y., Kong, Z., Zeng, T. et al. PARP1-siRNA suppresses human prostate cancer cell growth and progression. *Oncol Rep*. 10.3892/or.2018.6238. (Epub ahead of print 26 January 2018). 29393407

[147] Huang, M. et al. PARP1 negatively regulates transcription of BLM through its interaction with HSP90AB1 in prostate cancer. *J. Transl. Med.* **21**, 445 (2023). 37415147 10.1186/s12967-023-04288-z PMC10324254

[148] Li, H. et al. PARP1 inhibitor combined with Oxaliplatin efficiently suppresses Oxaliplatin resistance in gastric cancer-derived organoids via homologous recombination and the base excision repair pathway. *Front. Cell Dev. Biol.* 10.3389/fcell.2021.719192 (2021). 34497808 PMC8419238

[149] Zhu, X. et al. Circular RNA circATM binds PARP1 to suppress Wnt/β-catenin signaling and induce cell cycle arrest in gastric cancer cells. *J. Adv. Res.* 10.1016/j.jare.2025.04.033 (2026) (**Epub ahead of print 25 April 2025**). 40288674 PMC12869237

[150] Afzal, H. et al. *PARP1*: A potential biomarker for gastric cancer. *Pathol. Res. Pract.* **215**, 152472 (2019). 31174925 10.1016/j.prp.2019.152472

[151] Lanillos, J. et al. Interrogating the significance of *PARP1* expression and *PBRM1* mutation as biomarkers for predicting the response to Atezolizumab plus Bevacizumab or to Sunitinib in patients with Clear Cell Renal Cell Carcinoma. *Eur. Urol.* **82**, 334–335 (2022). 35688666 10.1016/j.eururo.2022.05.013

[152] Makhov, P. et al. Histone-dependent PARP-1 inhibitors: A novel therapeutic modality for the treatment of prostate and renal cancers. *Urol. Oncol.* **39**, 312–315 (2021). 32402770 10.1016/j.urolonc.2020.04.004 PMC9017175

[153] Hagiwara, M. et al. The significance of PARP1 as a biomarker for predicting the response to PD-L1 blockade in patients with *PBRM1*-mutated clear cell renal cell carcinoma. *Eur. Urol.* **81**, 145–148 (2022). 34627641 10.1016/j.eururo.2021.09.024

