# Supplementary material for: Computational discovery of emodin-based anthraquinones as PARP-1 inhibitors with relevance to ovarian and prostate cancer
Source: Sci Rep. 2026 Jun 21;16:28375. doi: 10.1038/s41598-026-55381-4 (PMC13562542; doi:10.1038/s41598-026-55381-4)
Supplement: Supplementary file 1 [file 41598_2026_55381_MOESM1_ESM.docx]

**Computational Discovery of Emodin-Based Anthraquinones as PARP-1 Inhibitors with Relevance to Ovarian and Prostate Cancer**

Supporting Information

Figure S1	2

Table S1	3

**Figure S1:** Three-dimensional ribbon structure of PARP-1 catalytic domain in complex with bound ligand.

**Table S1:** Molecular docking results of the selected ligands against the PARP-1 catalytic domain. The table summarizes the predicted binding affinities and docking scores of the ligands within the NAD⁺-binding pocket of PARP-1, providing a comparative assessment of their binding potential prior to molecular dynamics simulations.

| **BINDING AFFINITY (kcal/mol)** | | |
| --- | --- | --- |
| **Candidate Ligand** | **CID** | **7KK4** |
| L01 | 10086148 | -9.193 |
| L02 | 10207 | -9.329 |
| L03 | 10425624 | -10.03 |
| L04 | 10469084 | -9.24 |
| L05 | 10639 | -8.946 |
| L06 | 122635 | -9.026 |
| L07 | 122840 | -9.184 |
| L08 | 12313148 | -9.572 |
| L09 | 160717 | -9.082 |
| L10 | 318730 | -10.79 |
| L11 | 3220 | -9.233 |
| L12 | 343129 | -9.459 |
| L13 | 361515 | -8.997 |
| L14 | 626899 | -8.97 |
| L15 | 9866696 | -10 |
| Standard | 24958200 | -9.94 |
